# Supplementary material for: Altering the Sex Pheromone Cyclo(l-Pro-l-Pro) of the Diatom Seminavis robusta towards a Chemical Probe
Source: Int J Mol Sci. 2021 Jan 21;22(3):1037. doi: 10.3390/ijms22031037 (PMC7865345; doi:10.3390/ijms22031037)
Supplement: Supplementary file 1 [file ijms-22-01037-s001.pdf]

Supplementary Material

Altering the Sex Pheromone Cyclo(L-Pro-L-Pro) of the Diatom *Seminavis robusta* towards a Chemical Probe

Eli Bonneure [1], Amber De Baets [1], Sam De Decker [2], Koen Van den Berge [3], Lieven Clement [3], Wim Vyverman [2] and Sven Mangelinckx [1,*]

[1] Department of Green Chemistry and Technology – SynBioC, Faculty of Bioscience Engineering, Ghent University, Coupure Links 653, 9000 Ghent, Belgium.

[2] Department of Biology – Protistology and Aquatic Ecology, Faculty of Sciences, Ghent University, Krijgslaan 281/S8, 9000 Ghent, Belgium.

[3] Department of Applied Mathematics, Computer Science and Statistics, Faculty of Sciences, Ghent University, Krijgslaan 281/S9, 9000 Ghent, Belgium.

* Correspondence: Sven.Mangelinckx@UGent.be

Contents

S1 Diketopiperazine phytotoxicity **3**

S2 SIP⁻ containing medium **3**

S3 Interference assay | Threshold optimization **5**

S4 Interference assay | Diagram **7**

S5 Interference assay | Results **8**

S5.1 Glm results 8
S5.2 Calculation of the relative amount of attractive beads 10
S5.3 Error on the interference values 12

S6 Molecular properties **13**

S7 Syntheses **15**

S7.1 (2*S*,4*R*)-1-(*tert*-butoxycarbonyl)-4-hydroxypyrrolidine-2-carboxylic acid 10 15
S7.2 (*S*)-1-(*tert*-butoxycarbonyl)-4-oxopyrrolidine-2-carboxylic acid 11 16
S7.3 (*S*)-5-(*tert*-butoxycarbonyl)-1,2,5-triazaspiro[2.4]hept-1-ene-6-carboxylic acid 12 16
S7.4 Methyl (2*S*,4*R*)-4-hydroxypyrrolidine-2-carboxylate hydrochloride 13 17
S7.5 1-(*tert*-butyl) 2-methyl (2*S*,4*R*)-4-hydroxypyrrolidine-1,2-dicarboxylate 14 18
S7.6 1-(*tert*-butyl) 2-methyl (2*S*,4*R*)-4-((methylsulfonyl)oxy)pyrrolidine-1,2-dicarboxylate 15 18
S7.7 1-(*tert*-butyl) 2-methyl (2*S*,4*S*)-4-azidopyrrolidine-1,2-dicarboxylate 16 18
S7.8 Methyl (2*S*,4*S*)-4-azidopyrrolidine-2-carboxylate 2,2,2-trifluoroacetate 20 19
S7.9 1-(*tert*-butyl) 2-methyl (2*S*,4*S*)-4-hydroxypyrrolidine-1,2-dicarboxylate 17 19
S7.10 1-(*tert*-butyl) 2-methyl (2*S*,4*R*)-4-azidopyrrolidine-1,2-dicarboxylate 18 20
S7.11 Methyl (2*S*,4*R*)-4-azidopyrrolidine-2-carboxylate hydrochloride 24 20
S7.12 Methyl (2*S*,4*S*)-4-hydroxypyrrolidine-2-carboxylate hydrochloride 25 20
S7.13 Typical procedure for the synthesis of diketopiperazines: cyclo(L-Pro-L-Pro) 1 21

S8 NMR spectra of diketopiperazines. **23**

S8.1 Diketopiperazine 1 23
S8.2 Diketopiperazine 3 25
S8.3 Diketopiperazine 4 27
S8.4 Diketopiperazine 5 29
S8.5 Diketopiperazine 6 31
S8.6 Diketopiperazine 7 33
S8.7 Diketopiperazine 8 35
S8.8 Diketopiperazine 9 37

References **39**

S1 Diketopiperazine phytotoxicity

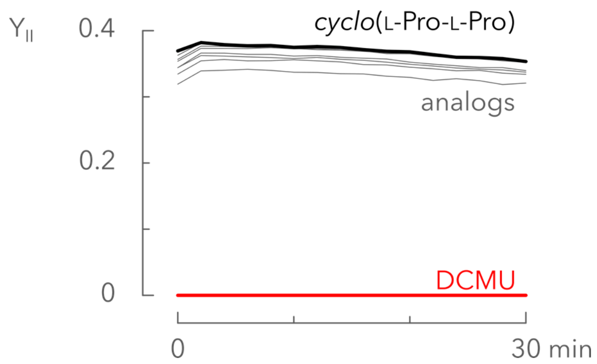

Figure S1 | Pulse amplitude modulation fluorometry data was used to assess pheromone phytotoxicity. The quantum yield of PS II (Y_{II}) is shown in the presence of pheromone *cyclo*(L-Pro-L-Pro) (**1**, 10 µM), DCMU (1 µM) and seven pheromone analogs (10 µM). The Y_{II}-values for DCMU were used as a reference for a complete inhibition of Y_{II}, those of the *cyclo*(L-Pro-L-Pro)–treatment as a reference of 0% inhibition. The inhibition values for the analogs were calculated through interpolation. The represented time-series are the average of five replicates. Measurements started five minutes after addition of the compounds and Y_{II} was determined every two minutes.

S2 SIP⁻ containing medium

Preparation

S. robusta 85B cultures were cultured in 12 cell culture flasks of 250 mL for one week and growth was monitored with PAM measurements (MAXI Imaging PAM M-series fluorometer, Walz Mess- und Regeltechnik, Effeltrich, Germany) and microscopic observations. The cultures were filtered when they reached the late-exponential phase, yielding 3 L of sterile spent medium. The medium was aliquoted in 50 mL falcons and stored at -20 °C.

Potency evaluation

The potency of the filtered medium was assessed using the interference assay described above. Cultures with 1 mL of ASW were conditioned with different dilutions of the filtered 85B medium: 1 mL aliquots of two dilutions (1:10 and 1:100) and an aliquot of undiluted spent medium were added to a 1 mL culture, resulting in three dilution factors (DF): 2, 20 and 200. To some wells, *cyclo*(L-Pro-L-Pro) **1** was added in a concentration of 100 nM. Assessing the attractiveness of the beads was carried out manually (threshold = 1) and the results are shown in Figure S2. As the effect of dilution was not significant, the filtered medium was used as a 1/100 dilution for every assay in this study.

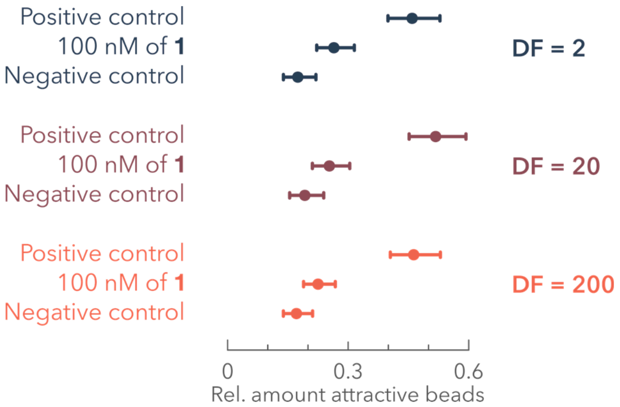

Figure S2 | The SIP⁻ containing medium can be diluted up to 200 times, without losing its potency. The estimated effect of the dilutions for the three treatments is not significant ($p > 0.05$). If the differences between the mutual treatments (positive control - 100 nM, 100 nM - negative control and positive - negative control) are considered, the dilution effect on these differences are also found to be not significant. The graph represents the actual attractiveness, with the mean ± 95% confidence interval ($n = 4$). DF = dilution factor.

S3 Interference assay | Threshold optimization

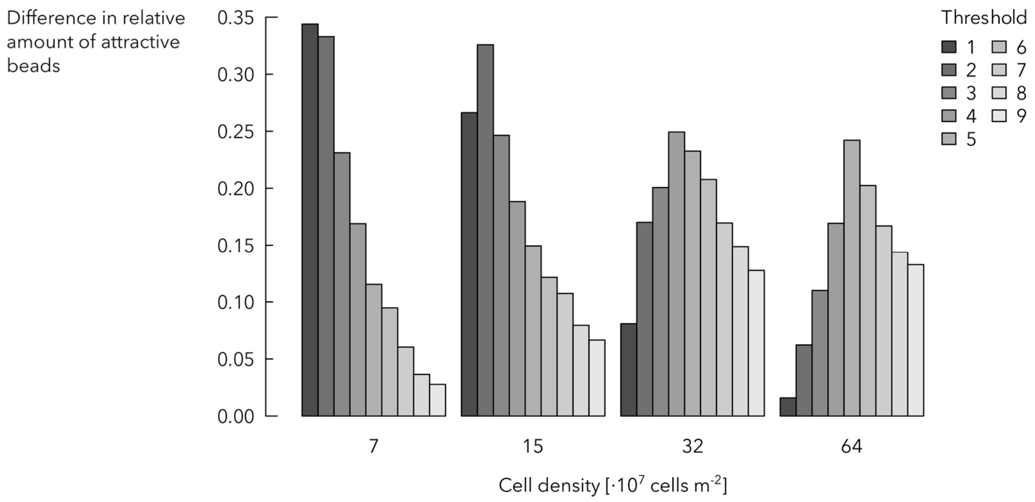

Figure S3. The optimal parameters for the interference assay are a density of 7 · 10^7 cells m^{-2} and a threshold of 1 cell $bead^{-1}$. Each bar represents the difference in relative attractivity towards pheromone-coated beads (2 nmol mg^{-1}) between the positive control and cultures treated with 10 μM *cyclo*(L-Pro-L-Pro) prior to bead addition ($n = 3$), with a given density and threshold. As can be seen on the graph, the threshold optimum is different for every density: the higher the density, the higher the optimal threshold is. The relative difference is maximal using a low density and a low threshold. The density of the two least dense cultures was determined after the medium renewal and before the dark-adaptation of the cultures by manual counting. The density of the remaining cultures was estimated by extrapolating the manual counts of the least dense cultures. The represented values are based on the difference between the estimated average attraction presented in Figure S4. The 95% confidence intervals of the differences are presented in Table S1.

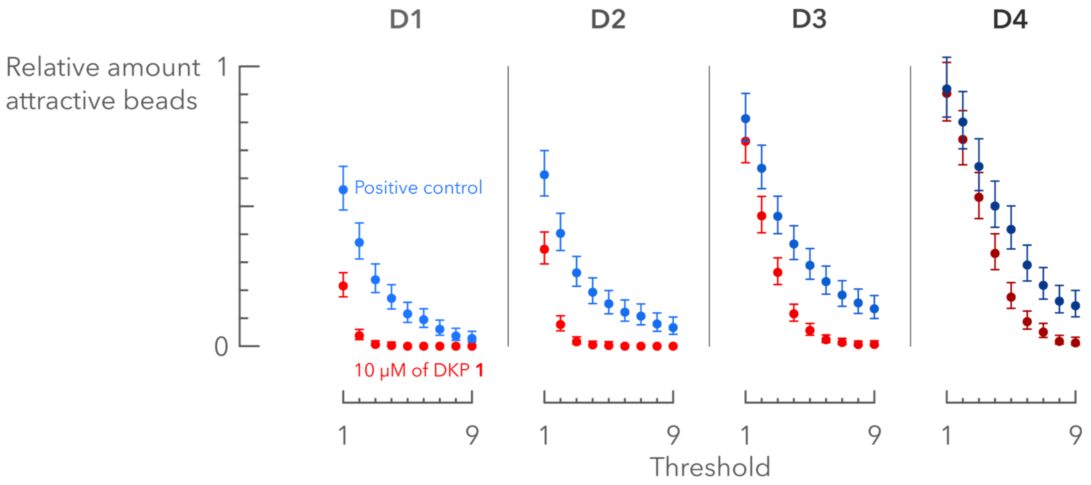

Figure S4 | Smaller threshold values yield a higher data variability. An interference test was set up to search for an optimum threshold and cell density. The test included two treatments, a positive control and 10 μM of *cyclo*(L-Pro-L-Pro) **1**, and four densities. The densities are 7·10^7 (D1), 15·10^7 (D2), 32·10^7 (D3) and 64·10^7 cells m^{-2} (D4). The error bars represent the 95% confidence interval, $n = 3$.

Table S1 | Threshold optimization: calculation of the 95% confidence intervals of the difference in relative fraction of attractive beads. The differences of the estimated average fraction of attractive beads between the positive control and **1** at 10 μM was calculated for every threshold and density. The error values of these differences were used to calculate the 95% c.i. of the estimated differences presented in Figure S3. D = density, T = threshold, E_{pos} = estimated average of the positive control, E_{DKP1} = estimated average of **1** at 10 μM. The original dataset contains zero values, resulting in large error values; zero-values were observed for entries #4–9,12–18,26–27.

	D	T	$ln(E_{pos})$	$ln(E_{DKP1})$	$ln(E_{pos})$-$ln(E_{DKP1})$	Std.Err[$ln(E_{pos})$-$ln(E_{DKP1})$]	95% c.i. of [$ln(E_{pos})$-$ln(E_{DKP1})$] lower	upper	E_{pos}	E_{DKP1}	95% c.i. of E_{pos} lower	upper	$E_{pos} - E_{DKP1}$	95% c.i. of [$E_{pos} - E_{DKP1}$] lower	upper
1	1	1	-5.81E-01	-1.54E+00	-9.55E-01	1.16E-01	-8.08E-01	-3.54E-01	0.55926	0.21529	0.44556	0.70196	0.34397	0.23027	0.48667
2	1	2	-9.93E-01	-3.28E+00	-2.29E+00	2.49E-01	-1.48E+00	-5.05E-01	0.37049	0.03754	0.22745	0.60349	0.33295	0.18991	0.56595
3	1	3	-1.44E+00	-5.08E+00	-3.64E+00	5.86E-01	-2.59E+00	-2.90E-01	0.23736	0.00620	0.07529	0.74833	0.23115	0.06908	0.74213
4	1	4	-1.77E+00	-6.20E+00	-4.43E+00	1.01E+00	-3.74E+00	2.07E-01	0.17099	0.00204	0.02377	1.22987	0.16896	0.02174	1.22783
5	1	5	-2.16E+00	-2.34E+01	-2.12E+01	3.29E+03	-6.45E+03	6.44E+03	0.11538	0.00000	0.00000	Inf	0.11538	0.00000	Inf
6	1	6	-2.36E+00	-2.44E+01	-2.20E+01	5.41E+03	-1.06E+04	1.06E+04	0.09478	0.00000	0.00000	Inf	0.09478	0.00000	Inf
7	1	7	-2.81E+00	-2.43E+01	-2.15E+01	5.43E+03	-1.06E+04	1.06E+04	0.06042	0.00000	0.00000	Inf	0.06042	0.00000	Inf
8	1	8	-3.31E+00	-2.33E+01	-2.00E+01	3.29E+03	-6.45E+03	6.44E+03	0.03647	0.00000	0.00000	Inf	0.03647	0.00000	Inf
9	1	9	-3.59E+00	-2.32E+01	-1.96E+01	3.28E+03	-6.42E+03	6.42E+03	0.02770	0.00000	0.00000	Inf	0.02770	0.00000	Inf
10	2	1	-4.90E-01	-1.06E+00	-5.70E-01	9.97E-02	-6.85E-01	-2.95E-01	0.61262	0.34633	0.50391	0.74480	0.26629	0.15758	0.39847
11	2	2	-9.09E-01	-2.56E+00	-1.65E+00	1.87E-01	-1.28E+00	-5.42E-01	0.40306	0.07724	0.27928	0.58170	0.32582	0.20205	0.50446
12	2	3	-1.34E+00	-4.15E+00	-2.81E+00	3.89E-01	-2.10E+00	-5.76E-01	0.26220	0.01579	0.12229	0.56217	0.24641	0.10650	0.54638
13	2	4	-1.65E+00	-5.41E+00	-3.77E+00	7.15E-01	-3.05E+00	-2.44E-01	0.19282	0.00446	0.04747	0.78330	0.18836	0.04301	0.77884
14	2	5	-1.89E+00	-6.09E+00	-4.21E+00	1.01E+00	-3.86E+00	8.87E-02	0.15171	0.00226	0.02106	1.09277	0.14945	0.01880	1.09051
15	2	6	-2.11E+00	-2.43E+01	-2.22E+01	5.36E+03	-1.05E+04	1.05E+04	0.12156	0.00000	0.00000	Inf	0.12156	0.00000	Inf
16	2	7	-2.23E+00	-2.42E+01	-2.20E+01	5.36E+03	-1.05E+04	1.05E+04	0.10738	0.00000	0.00000	Inf	0.10738	0.00000	Inf
17	2	8	-2.53E+00	-2.32E+01	-2.06E+01	3.23E+03	-6.34E+03	6.34E+03	0.07946	0.00000	0.00000	Inf	0.07946	0.00000	Inf
18	2	9	-2.71E+00	-2.31E+01	-2.04E+01	3.20E+03	-6.27E+03	6.27E+03	0.06657	0.00000	0.00000	Inf	0.06657	0.00000	Inf
19	3	1	-2.07E-01	-3.11E-01	-1.05E-01	6.72E-02	-3.38E-01	-7.50E-02	0.81330	0.73241	0.71298	0.92774	0.08089	-0.01943	0.19533
20	3	2	-4.53E-01	-7.64E-01	-3.11E-01	8.17E-02	-6.13E-01	-2.93E-01	0.63587	0.46573	0.54179	0.74627	0.17014	0.07607	0.28055
21	3	3	-7.67E-01	-1.33E+00	-5.66E-01	1.03E-01	-9.68E-01	-5.66E-01	0.46432	0.26369	0.37969	0.56780	0.20062	0.11600	0.30410
22	3	4	-1.01E+00	-2.16E+00	-1.15E+00	1.41E-01	-1.28E+00	-7.32E-01	0.36514	0.11577	0.27719	0.48100	0.24937	0.16142	0.36523
23	3	5	-1.24E+00	-2.88E+00	-1.64E+00	1.94E-01	-1.62E+00	-8.62E-01	0.28891	0.05632	0.19768	0.42223	0.23259	0.14136	0.36591
24	3	6	-1.47E+00	-3.77E+00	-2.31E+00	2.91E-01	-2.04E+00	-8.96E-01	0.23070	0.02299	0.13039	0.40821	0.20771	0.10739	0.38521
25	3	7	-1.70E+00	-4.33E+00	-2.63E+00	3.92E-01	-2.47E+00	-9.32E-01	0.18271	0.01313	0.08481	0.39360	0.16958	0.07169	0.38047
26	3	8	-1.87E+00	-5.15E+00	-3.28E+00	5.88E-01	-3.02E+00	-7.14E-01	0.15459	0.00580	0.04880	0.48967	0.14879	0.04300	0.48387
27	3	9	-2.01E+00	-5.09E+00	-3.08E+00	5.91E-01	-3.17E+00	-8.54E-01	0.13376	0.00613	0.04204	0.42563	0.12763	0.03590	0.41950
28	4	1	-8.39E-02	-1.01E-01	-1.74E-02	7.72E-02	-2.35E-01	6.74E-02	0.91948	0.90360	0.79034	1.06971	0.01588	-0.11325	0.16611
29	4	2	-2.22E-01	-3.03E-01	-8.09E-02	8.51E-02	-3.89E-01	-5.49E-02	0.80113	0.73885	0.67805	0.94656	0.06229	-0.06080	0.20771
30	4	3	-4.43E-01	-6.31E-01	-1.88E-01	9.72E-02	-6.33E-01	-2.53E-01	0.64211	0.53206	0.53078	0.77679	0.11005	-0.00129	0.24473
31	4	4	-6.92E-01	-1.10E+00	-4.13E-01	1.16E-01	-9.19E-01	-4.64E-01	0.50081	0.33152	0.39888	0.62879	0.16930	0.06737	0.29728
32	4	5	-8.74E-01	-1.74E+00	-8.68E-01	1.48E-01	-1.16E+00	-5.83E-01	0.41738	0.17526	0.31213	0.55814	0.24212	0.13686	0.38287
33	4	6	-1.24E+00	-2.43E+00	-1.20E+00	2.00E-01	-1.63E+00	-8.45E-01	0.29011	0.08767	0.19589	0.42965	0.20243	0.10821	0.34198
34	4	7	-1.53E+00	-2.99E+00	-1.46E+00	2.63E-01	-2.04E+00	-1.01E+00	0.21742	0.05046	0.12978	0.36425	0.16697	0.07932	0.31379
35	4	8	-1.83E+00	-4.07E+00	-2.24E+00	4.31E-01	-2.67E+00	-9.83E-01	0.16083	0.01713	0.06912	0.37418	0.14369	0.05199	0.35705
36	4	9	-1.93E+00	-4.44E+00	-2.50E+00	5.21E-01	-2.96E+00	-9.12E-01	0.14456	0.01184	0.05203	0.40164	0.13272	0.04019	0.38980

S4 Interference assay | Diagram

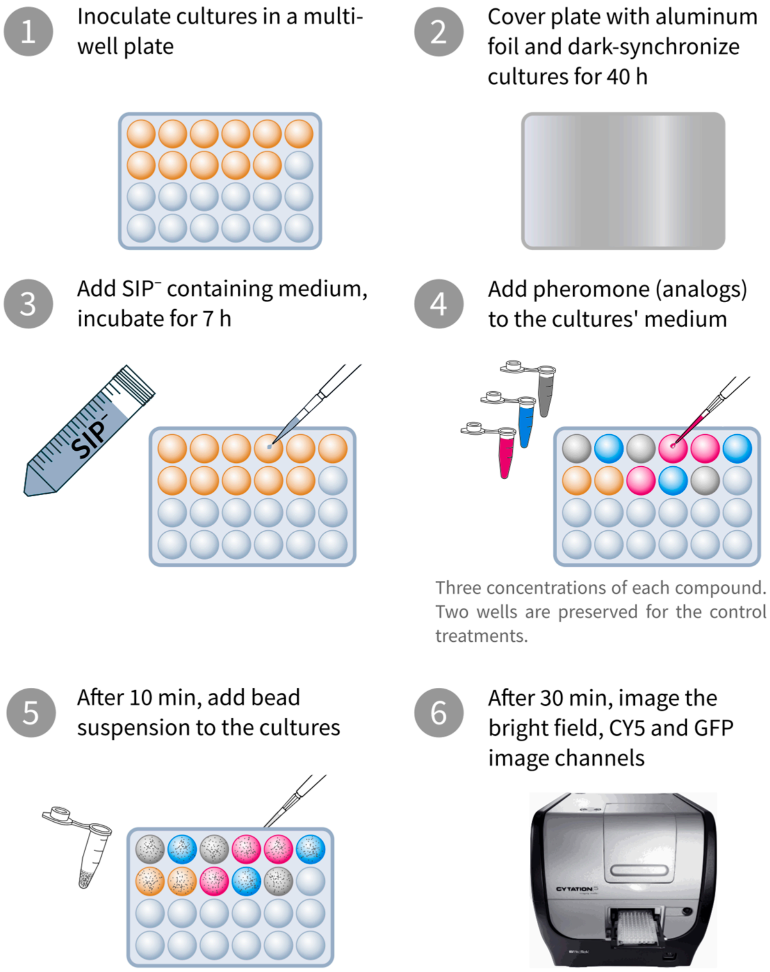

Figure S5 | A diagram of a typical interference assay. Every 24-well plate represents one replicate and the treatments are randomly assigned.

S5 Interference assay | Results

S5.1 Glm results

Table S2 | Glm results from Experiment A (family = poisson)

	Estimate	*Std. Error*	*z value*	*Pr(>|z|)*	
(Intercept)	-0.73186	0.07421	-9.863	< 2e-16	***
negative_control	-0.63562	0.11428	-5.562	2.67E-08	***
DKP1_10nM	-0.21713	0.10114	-2.147	0.031807	*
DKP1_100nM	-0.29	0.10175	-2.85	0.004372	**
DKP1_10µM	-0.36834	0.10211	-3.607	0.000309	***
DKP3_10nM	-0.06813	0.09619	-0.708	0.478782	
DKP3_100nM	-0.33622	0.10434	-3.223	0.001271	**
DKP3_10µM	-0.38207	0.10178	-3.754	0.000174	***
DKP7_10nM	-0.23834	0.10248	-2.326	0.02003	*
DKP7_100nM	-0.13481	0.09615	-1.402	0.160876	
DKP7_10µM	-0.33919	0.10515	-3.226	0.001257	**
plate2	-0.12494	0.05824	-2.145	0.031927	*
plate3	0.17744	0.05394	3.29	0.001003	**

Table S3 | Glm results from Experiment B (family = poisson)

	Estimate	*Std. Error*	*z value*	*Pr(>|z|)*	
(Intercept)	-0.67106	0.12769	-5.255	1.48E-07	***
negative_control	-1.26614	0.22271	-5.685	1.31E-08	***
DKP1_10nM	-0.64547	0.16611	-3.886	0.000102	***
DKP1_100nM	-0.68057	0.17118	-3.976	7.01E-05	***
DKP1_10µM	-1.00957	0.18838	-5.359	8.36E-08	***
DKP4_10nM	-0.1211	0.14501	-0.835	0.403633	
DKP4_100nM	-0.76011	0.17664	-4.303	1.68E-05	***
DKP4_10µM	-0.84503	0.17306	-4.883	1.05E-06	***
DKP8_10nM	-0.25205	0.15057	-1.674	0.094152	.
DKP8_100nM	-0.08317	0.13762	-0.604	0.545634	
DKP8_10µM	-1.10792	0.1934	-5.728	1.01E-08	***
plate2	-0.00283	0.10699	-0.026	0.978895	
plate3	-0.42923	0.12645	-3.394	0.000688	***
plate4	-0.52179	0.13435	-3.884	0.000103	***
plate5	-0.40911	0.12596	-3.248	0.001162	**
plate6	-0.3794	0.12697	-2.988	0.002808	**

Table S4 | Glm results of Experiment C (family = poisson)

	Estimate	*Std. Error*	*z value*	*Pr(>|z|)*	
(Intercept)	-0.87986	0.13602	-6.469	9.88E-11	***
negative_control	-0.87825	0.2297	-3.823	0.000132	***
DKP1_10nM	-0.57323	0.20688	-2.771	0.005592	**
DKP1_100nM	-0.71868	0.24433	-2.941	0.003267	**

DKP1_10μM	-0.36565	0.20518	-1.782	0.074736	.
DKP6_10nM	-0.02542	0.16816	-0.151	0.879852	
DKP6_100nM	-0.09386	0.17405	-0.539	0.589702	
DKP6_10μM	-0.70199	0.21791	-3.222	0.001275	**
DKP9_10nM	-0.09393	0.18288	-0.514	0.607521	
DKP9_100nM	-0.28237	0.19649	-1.437	0.150705	
DKP9_10μM	-0.42877	0.18814	-2.279	0.022664	*
DKP5_10nM	-0.37764	0.19948	-1.893	0.058345	.
DKP5_100nM	-0.40244	0.19506	-2.063	0.039101	*
DKP5_10μM	-0.43391	0.20909	-2.075	0.037962	*
plate2	0.28539	0.10188	2.801	0.005089	**
plate5	0.10054	0.14532	0.692	0.489017	
plate6	0.2502	0.11214	2.231	0.02567	*

S5.2 Calculation of the relative amount of attractive beads

E = Relative amount attractive beads

Table S5 | Calculated interference values for experiment A

		ln(E)	*Std. Err[ln(E)]*	*95% c.i. of ln(E)*		*E*	*95% c.i. of E*		*Interference**
				lower	*upper*		*lower*	*upper*	
1	Positive control	-0.73186	0.07421	-0.58642	-0.87730	0.48101	0.41590	0.55632	0.00
2	Negative control	-1.36748	0.09772	-1.17596	-1.55900	0.25475	0.21035	0.30852	1.87
3	DKP1_10nM	-0.94899	0.08222	-0.78784	-1.11014	0.38713	0.32951	0.45483	0.78
4	DKP1_100nM	-1.02186	0.08318	-0.85883	-1.18489	0.35993	0.30578	0.42366	1.00
5	DKP1_10µM	-1.10020	0.08298	-0.93757	-1.26284	0.33280	0.28285	0.39158	1.22
6	DKP3_10nM	-0.79999	0.07583	-0.65136	-0.94862	0.44933	0.38727	0.52134	0.26
7	DKP3_100nM	-1.06808	0.08495	-0.90158	-1.23459	0.34367	0.29095	0.40593	1.13
8	DKP3_10µM	-1.11393	0.08364	-0.94999	-1.27786	0.32827	0.27863	0.38674	1.26
9	DKP7_10nM	-0.97020	0.08452	-0.80455	-1.13585	0.37901	0.32115	0.44729	0.84
10	DKP7_100nM	-0.86667	0.07571	-0.71827	-1.01507	0.42035	0.36238	0.48759	0.50
11	DKP7_10µM	-1.07105	0.08632	-0.90187	-1.24023	0.34265	0.28932	0.40581	1.14

* relative to DKP1_100 nM

Table S6 | Calculated interference values for experiment B

		ln(E)	*Std. Err [ln(E)]*	*95% c.i. of ln(E)*		*E*	*95% c.i. of E*		*Interference**
				lower	*upper*		*lower*	*upper*	
1	Positive control	-0.67106	0.12769	-0.92132	-0.42079	0.51117	0.39799	0.65653	0.00
2	Negative control	-1.93719	0.20621	-2.34136	-1.53303	0.14411	0.09620	0.21588	1.45
3	DKP1_10nM	-1.31653	0.14176	-1.59438	-1.03868	0.26806	0.20303	0.35392	0.96
4	DKP1_100nM	-1.35163	0.15026	-1.64614	-1.05712	0.25882	0.19279	0.34746	1.00
5	DKP1_10µM	-1.68063	0.16856	-2.01100	-1.35026	0.18626	0.13386	0.25917	1.29
6	DKP4_10nM	-0.79216	0.11898	-1.02536	-0.55896	0.45287	0.35867	0.57180	0.23

7	DKP4_100nM	-1.43116	0.15366	-1.73234	-1.12999	0.23903	0.17687	0.32304	1.08
8	DKP4_10µM	-1.51609	0.15306	-1.81608	-1.21610	0.21957	0.16266	0.29638	1.16
9	DKP8_10nM	-0.92310	0.12868	-1.17530	-0.67090	0.39728	0.30872	0.51125	0.45
10	DKP8_100nM	-0.75422	0.11069	-0.97118	-0.53727	0.47038	0.37864	0.58434	0.16
11	DKP8_10µM	-1.77897	0.17423	-2.12045	-1.43749	0.16881	0.11998	0.23752	1.36

* relative to DKP1_100 nM

Table S7 | Calculated interference values of experiment C

		ln(E)	*Std. Err[ln(E)]*	*95% c.i. of ln(E)*		*E*	*95% c.i. of E*		*Interference**
				lower	***upper***		***lower***	***upper***	
1	Positive control	-0.87986	0.13602	-1.14645	-0.61327	0.41484	0.31776	0.54158	0.00
2	Negative control	-1.75811	0.20985	-2.16940	-1.34682	0.17237	0.11425	0.26007	1.14
3	DKP1_10nM	-1.45309	0.17941	-1.80474	-1.10145	0.23385	0.16452	0.33239	0.85
4	DKP1_100nM	-1.59854	0.22264	-2.03492	-1.16217	0.20219	0.13069	0.31281	1.00
5	DKP1_10µM	-1.24551	0.18149	-1.60124	-0.88979	0.28779	0.20165	0.41074	0.60
6	DKP6_10nM	-0.90528	0.13288	-1.16572	-0.64484	0.40443	0.31170	0.52475	0.05
7	DKP6_100nM	-0.97372	0.14255	-1.25312	-0.69433	0.37767	0.28561	0.49941	0.17
8	DKP6_10µM	-1.58185	0.19326	-1.96064	-1.20306	0.20559	0.14077	0.30027	0.98
9	DKP9_10nM	-0.97379	0.15199	-1.27168	-0.67590	0.37765	0.28036	0.50870	0.17
10	DKP9_100nM	-1.16223	0.16862	-1.49273	-0.83174	0.31279	0.22476	0.43529	0.48
11	DKP9_10µM	-1.30863	0.16271	-1.62753	-0.98973	0.27019	0.19641	0.37168	0.68
12	DKP5_10nM	-1.25750	0.17160	-1.59382	-0.92118	0.28436	0.20315	0.39805	0.61
13	DKP5_100nM	-1.28230	0.16373	-1.60320	-0.96140	0.27740	0.20125	0.38236	0.65
14	DKP5_10µM	-1.31378	0.18115	-1.66883	-0.95872	0.26880	0.18847	0.38338	0.69

* relative to DKP1_100 nM

S5.3 Error on the interference values

Table S8 | Calculation of the 95% confidence interval of the interference values. The differences of the estimated average fraction of attractive beads between the synthetic analogs and **1** were calculated. The error values of these differences were used to calculate the 95% c.i. of the estimated fraction attractive beads (ln(E)). The estimated average interference values and confidence intervals were calculated relative to the difference of **1** at 100 nM and the positive control (see section S5.2).

	Experiment and treatment		*ln(E)*	*ln(E)-ln(E_{DKP1})*	*Std.Err[ln(E)-ln(E_{DKP1})]*	*95% c.i. of [ln(E)-ln(E_{DKP1})]*		*95% c.i. of ln(E)*		*95% c.i. of E*		*Interference*	*95% c.i. of interference*	
						lower	*upper*	*lower*	*upper*	*lower*	*upper*		*lower*	*upper*
1	A	DKP1_100nM	-1.02186									1.00		
2	A	DKP3_100nM	-1.06808	-0.04622	0.11061	-0.26302	0.17057	-1.28488	-0.85128	0.27668	0.42687	1.13	0.45	1.69
3	A	DKP7_100nM	-0.86667	0.15519	0.10290	-0.04650	0.35687	-1.06836	-0.66498	0.34357	0.51428	0.50	-0.27	1.14
4	B	DKP1_100nM	-1.35163									1.00		
5	B	DKP4_100nM	-1.43116	-0.07953	0.19580	-0.46330	0.30423	-1.81492	-1.04740	0.35085	1.07841	1.08	0.64	1.38
6	B	DKP8_100nM	-0.75422	0.59740	0.16190	0.28009	0.91472	-1.07154	-0.43691	0.64603	0.16165	0.16	-0.53	0.67
7	C	DKP1_100nM	-1.59854									1.00		
8	C	DKP6_100nM	-0.97372	0.62482	0.24840	0.13796	1.11168	-1.46058	-0.48686	0.61455	0.17478	0.17	-0.94	0.86
9	C	DKP9_100nM	-1.16223	0.43631	0.26482	-0.08273	0.95535	-1.68128	-0.64319	0.52561	0.47991	0.48	-0.52	1.08
10	C	DKP5_100nM	-1.28230	0.31624	0.26356	-0.20032	0.83281	-1.79887	-0.76573	0.46499	0.64633	0.65	-0.24	1.17

S6 Molecular properties

Table S9 gives an overview of the calculated molecular properties of the compounds presented in this study. The molecular volumes and the LogP values of the compounds were calculated using MarvinView 18.22.0 after geometry optimization (lowest energy conformer with the Dreiding force field). The relative molecular volume shows the molecular volume relative to diketopiperazine **1**.

The $\Delta\delta_{\beta\gamma}$ values represent the difference between δC^{β} and δC^{γ} values. The corresponding dihedral angles θ (O=C–C$^{\alpha}$–C$^{\beta}$) were calculated with the empirical relationship $\Delta\delta_{\beta\gamma} = 0.081 \cdot |\theta| + 2.47$ as described by Siemion et al.[1]

Table S9 | Overview of the calculated molecular properties of the diketopiperazines presented in this study.

	Compound	***Molecular volume [Å³]***	***Relative molecular volume***	***δC^{β}***	***δC^{γ}***	***$\Delta\delta_{\beta\gamma}$***	***ϑ [°]***	***LogP***
1	DKP **1**	175.28	1.00	27.7	23.4	4.3	22.6	-0.528
2	DKP **3**	181.29	1.03	28.0	23.1	4.9	30.0	-0.422
3	DKP **4**	198.75	1.13	27.6	23.4	4.2	21.4	0.412
4	DKP **5**	198.88	1.13	27.6	23.3	4.3	22.6	0.412
5	DKP **6**	183.91	1.05	27.5	23.4	4.1	20.1	-1.675
6	DKP **7**	201.54	1.15	27.7	23.3	4.4	23.8	-1.032
7	DKP **8**	227.95	1.30	27.7	23.3	4.4	23.8	-0.368
8	DKP **9**	158.24	0.90	26.9	23.2	3.7	15.2	-1.045

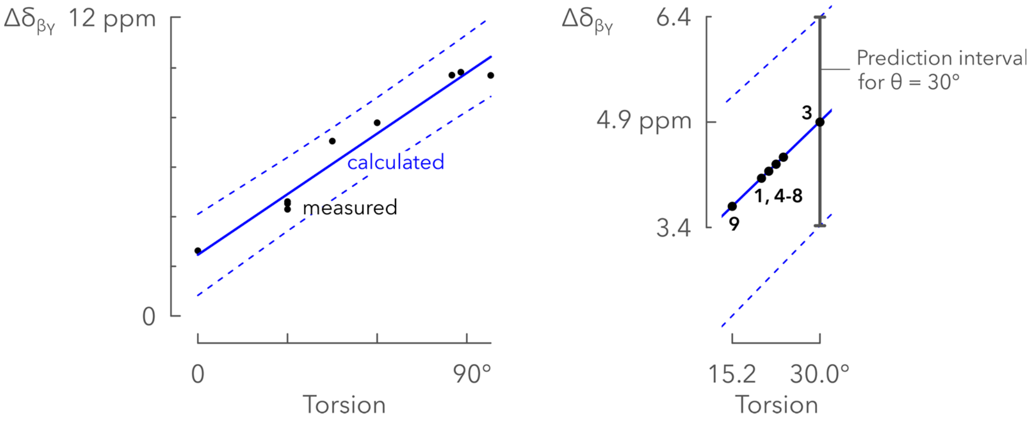

Figure S6 | (left) Linear relationship between the torsion (θ) and the difference between the ^{13}C signals of C_{β} and C_{γ} ($\Delta\delta_{\beta\gamma}$). The dashed line represents the prediction interval of the fit (solid line). Data from Siemion et al.[1] **(right)** The calculated torsion values of diketopiperazines **1** and **3-9** all fall within the prediction interval for a boat conformation (θ = 30°).

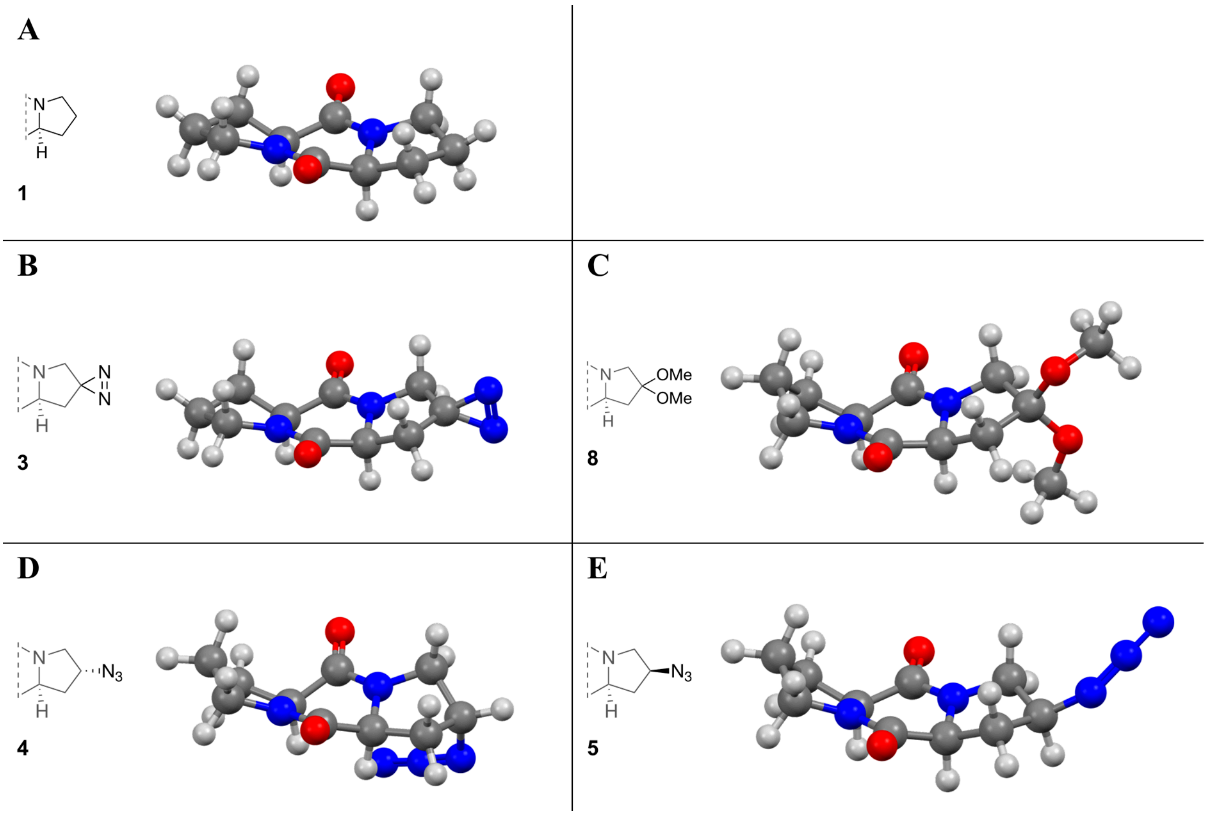

Figure S7 | Crystal structure of cyclo(L-Pro-L-Pro) 1 (A) and modelled structures of pheromone analogs (B-E). The crystal structure of the pheromone was published by Benedetti et al.[2] The diketopiperazine was reported to have a nearly planar structure. Figures **B** and **C** illustrate the volumetric difference between diazirine **3** and acetal **8**. The azide moiety in the pseudo-axial position (**D**) faces the convex side of the molecule, while the pseudo-equatorial azide (**E**) is positioned in the same plane as the diketopiperazine scaffold. Structures **B** to **E** were modeled using Chem3D (MM2).

S7 Syntheses

Solvents and commercially available reagents were obtained from Sigma-Aldrich (Missouri, USA), ChemPur (Karlsruhe, Germany), Acros (Geel, Belgium), Alfa Aesar (Ward Hill MA, USA), TCI Chemicals (Tokyo, Japan) and Air Liquide (Paris, France). All solvents were used without further purification. DMF was dried over activated molecular sieves for at least 48 h, THF was dried using an MBRAUN SPS-800 solvent purification system and dry methanol on molecular sieves was purchased from Acros. Boc anhydride was heated to its melting temperature prior to application. All reported temperatures were measured externally.

Reversed phase chromatography was carried out using a Grace Reveleris™ Flash Chromatography system with reusable columns (C18, 20-40 µm, 4 g to 120 g depending on sample size). Infrared spectra were recorded on a shimadzu IRAFFINITY-1S Fourier Transform Infrared Spectrophotometer (FTIR). Melting points were determined using a Wagner and Munz Kofler WME. Optical rotations were measured with a JASCO P-2000 series polarimeter. Specific rotations are reported with the standard deviations of the measurements (n = 10), concentrations are reported in grams per 100 mL. High resolution mass spectrometry (HRMS) was performed on an Agilent 6220 TOF mass spectrometer equipped with an ESI/APCI-multimode source.

All synthesized pheromone analogs **3** to **9** were purified with a preparative HPLC prior to biological evaluation. The equipment used consisted of an Agilent 1100 Series system (Agilent, Santa Clara CA, USA) with a Zorbax Eclipse XDB-C18 column (21.2 mm x 150 mm, 5 µm, Agilent) or a Supelco Ascentis C18 column (21.2 mm x 150 mm, 5 µm, Sigma-Aldrich).

All ^{1}H and ^{13}C NMR spectra were recorded at 400 and 100.6 MHz respectively, on a Bruker Avance III, equipped with ^{1}H/BB z-gradient probe (BBO, 5 mm). All spectra were processed using Topspin 3.2. ^{1}H, ^{13}C, COSY, HSQC, HMBC and APT spectra were acquired through the standard sequences available in the Bruker pulse program library. In all reported spectra $CDCl_3$ and TMS were used as solvent and internal standard, respectively. The spectra were analyzed using Topspin 3.5.

S7.1 (2*S*,4*R*)-1-(*tert*-butoxycarbonyl)-4-hydroxypyrrolidine-2-carboxylic acid 10

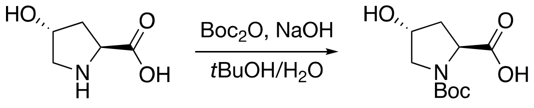

2 **10**

Trans-4-hydroxy-L-proline **2** (6.56 g, 50 mmol) was dissolved in 60 mL of a 0.9 M NaOH solution and the mixture was diluted with 38 mL *tert*-butanol. Next, 11.5 mL Boc anhydride was added dropwise (10.91 g, 50 mmol, 1 equiv.) and the reaction mixture was stirred for 16 h. The reaction mixture was extracted with petroleum ether (2 x 15 mL) and the combined organic fractions were extracted twice with 5 mL of a saturated $NaHCO_3$ solution. The combined aqueous phases were acidified until pH 1 to 1.5 at 0 °C with a 1.1 M $KHSO_4$ solution and extracted with ethyl acetate (4 x 20 mL). The combined organic phases were washed with 10 mL of water and dried with $MgSO_4$. Removal of the solvents *in vacuo* yielded a yellowish viscous oil with a mass of 10.3 g (89%). The product was used without further purification. Spectral data were in accordance with literature.[3]

S7.2 (*S*)-1-(*tert*-butoxycarbonyl)-4-oxopyrrolidine-2-carboxylic acid 11

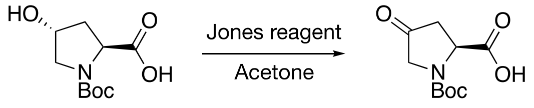

Hydroxyproline **10** (3.1 g, 13.4 mmol) was dissolved in 250 mL acetone and cooled to 0 °C. Freshly prepared Jones reagent (25.5 mL, 2 M CrO_3, 15% H_2SO_4, 3.8 equiv.) was added dropwise to the cooled solution over 25 minutes. The reaction was stirred for 20 minutes at 0 °C, after which the ice bath was removed. The reaction was stirred for another 3 h and stopped by dropwise addition of 20 mL isopropanol. During the addition of isopropanol, the mixture was cooled with a cold-water bath. After 30 minutes, the solution was filtered over Celite® and the filtrate was concentrated with a rotary evaporator. The resulting slurry was diluted with 500 mL ethyl acetate and the solution was again filtered over Celite®. The resulting filtrate was washed with brine (6 x 200 mL) and the organic phase was dried with $MgSO_4$. Recrystallization of the resulting solids in ethyl acetate, yielded 1.54 g (50%) of white crystals. The spectral data were in accordance with literature.[4]

S7.3 (*S*)-5-(*tert*-butoxycarbonyl)-1,2,5-triazaspiro[2.4]hept-1-ene-6-carboxylic acid 12

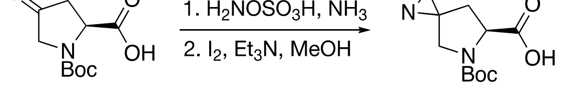

The synthesis of diazirine **12** was adopted from Van der Meijden et al.[4] About 100 mL ammonia was condensed in a three-necked round-bottom flask containing oxoproline **11** (2.72 g, 12 mmol) and the solution was refluxed for 5 h. A suspension of 1.475 g

hydroxylamine-O-sulfonic acid (13 mmol, 1.1 equiv.) in 7 mL of dry methanol at 12 °C (dry ice/dioxane bath) was added dropwise to the reaction mixture. The reaction mixture was refluxed for an extra 1.5 h and 15 mL dry methanol was added afterwards. The condenser was replaced by a cotton plug and the mixture was stirred for 16 h, allowing the ammonia to evaporate. The resulting slurry was filtered over a glass frit filter and the filter cake was washed with methanol (2 x 35 mL). The combined methanol phases are treated with 1.65 mL Et_3N (11.9 mmol, 1 equiv.) and the resulting solution was concentrated to less than 15 mL. The concentrated solution was again treated with one equivalent Et_3N, cooled to 0 °C with an ice bath and titrated with a freshly prepared 0.1 M solution of iodine in methanol until the solution retained an orange color. The solvent was removed *in vacuo* and the resulting slurry was dissolved in 50 mL water. The pH of the solution was adjusted to 2 and the diazirine was extracted with ethyl acetate (4 x 35 mL). The combined organic phases were washed once with brine (15 mL) and are dried over $MgSO_4$. After removal of the solvent *in vacuo*, the diazirine was purified with reversed phase chromatography (ACN/water, 5/95 to 10/90 during 10 CV, 24/76 during 10 CV and 24/76 to 50/50 during 10 CV) yielding 0.884 g (31%) of yellow crystals. Spectral data of the ^{13}C spectrum did not converge with the literature source.

^{1}H NMR (400 MHz, $CDCl_3$, δ): 1.47 (9H, s, $C(CH_3)_3$), 1.61-1.75 (1H, m, CH(*H*CH)), 2.29-2.42 (1H, m, CH(HC*H*)), 3.06-3.27 (2H, m, NCH_2), 4.56 + 4.66 (1H, 2 x d, J = 8.3 Hz, CH) and 9.58 (1H, br. s, COOH).

^{13}C NMR (100.6 MHz, $CDCl_3$, δ): 28.3 + 28.4 (CH_3), 30.4 + 30.9 (CN_2), 32.5 + 33.7 ($CHCH_2$), 48.3 + 48.6 (NCH_2), 57.8 + 58.0 (CH), 81.6 + 81.9 ($C(CH_3)_3$), 153.5 + 154.8 (NCO) and 176.0 + 177.6 (COOH).

S7.4 Methyl (2*S*,4*R*)-4-hydroxypyrrolidine-2-carboxylate hydrochloride 13

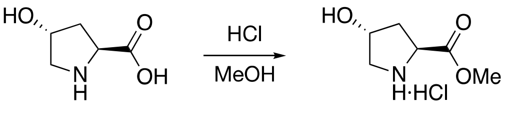

2 13

A round-bottom flask containing 400 mL dry MeOH was cooled to 0 °C. Acetyl chloride (40.8 mL, 57.2 mmol, 1.5 equiv.) was added dropwise and the mixture was allowed to react for 20 minutes, after which 50 g hydroxyproline **2** was added (38.1 mmol). The reaction mixture was refluxed for 16 h, cooled to 20 °C and poured in 830 mL diethyl ether. After filtration, the precipitate was washed with diethyl ether and dried to the air, yielding 62.0 g of white crystals (90%). Spectral data in accordance with literature.[5]

S7.5 1-(*tert*-butyl) 2-methyl (2*S*,4*R*)-4-hydroxypyrrolidine-1,2-dicarboxylate 14

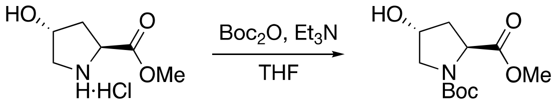

13 **14** f

Hydroxyproline **13** (10 g, 55 mmol) was dissolved in 300 mL 1,4-dioxane. 15.2 mL Boc anhydride (14.4 g, 66 mmol, 1.2 equiv.) was added dropwise. 210 mL of a saturated $NaHCO_3$ solution was added and the reaction mixture was stirred for 16 h. Dichloromethane (200 mL) was added and the organic phase was washed with water (2 x 40 mL) and brine (40 mL). The organic phase was dried with $MgSO_4$ and the solvent was removed *in vacuo*. 12.3 g of a white solid was obtained (79%). Spectral data were in accordance with literature.[6]

S7.6 1-(*tert*-butyl) 2-methyl (2*S*,4*R*)-4-((methylsulfonyl)oxy)pyrrolidine-1,2-dicarboxylate 15

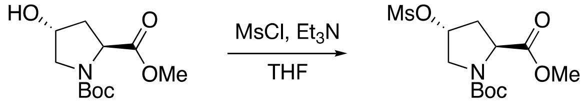

14 **15**

Hydroxyproline **14** (7.22 g, 29.4 mmol) was dissolved in 150 mL dry THF and cooled to 0 °C. Triethylamine (4.92 mL, 35 mmol, 1.2 equiv.) was added, followed by a dropwise addition of methanesulfonyl chloride (2.51 mL, 32.4 mmol, 1.1 equiv.) and stirred for 16 h at 20 °C. Afterwards, the mixture was diluted with 300 mL THF and the organic solvent was washed with 5% HCl, 2% Na_2CO_3 and brine (30 mL each). The organic phase was dried with $MgSO_4$ and the solvent was removed *in vacuo*. After purification with flash chromatography (petroleum ether/ethyl acetate, 3:1, R_f = 0.14), 4.04 g (42%) of light brown crystals were obtained. Spectra were in accordance with literature.[7]

S7.7 1-(*tert*-butyl) 2-methyl (2*S*,4*S*)-4-azidopyrrolidine-1,2-dicarboxylate 16

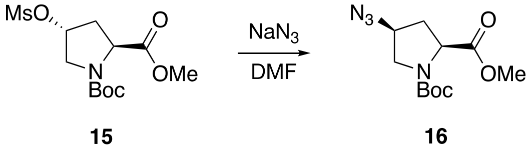

15 **16**

Hydroxyproline derivative **15** (2.5 g, 7.7 mmol) was dissolved in in 40 mL dry DMF under nitrogen atmosphere and NaN_3 (2.01 g, 31 mmol, 4 equiv.) was added. The reaction mixture was stirred for 16 h at 70 °C, after which the mixture was poured out in 100 mL ice water. The aqueous phase was extracted with ethyl acetate (3 x 100 mL) and the combined organic phases were washed with water (50 mL) and a 1 M LiCl solution (3 x 35 mL). The organic phase was

dried with $MgSO_4$ and removal of the solvent *in vacuo* yielded 1.97 g (94%) of product as a yellow oil. Spectral data were in accordance with literature.[7]

S7.8 Methyl (2*S*,4*S*)-4-azidopyrrolidine-2-carboxylate 2,2,2-trifluoroacetate 20

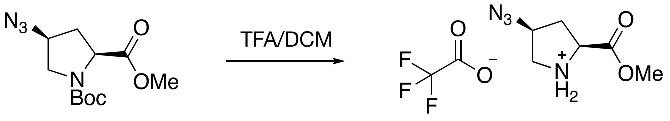

16 **19**

Azidoproline **16** (1.97 g, 7.3 mmol) was dissolved in 10 mL DCM. The solution was cooled to 0 °C and 10 mL TFA was added. The reaction mixture was stirred for 1 h, after which the TFA was removed azeotropically with toluene (5 x 50 mL). The product was obtained as a dark oil in a quantitative yield, used without further purification. Spectral data were in accordance with literature.[8]

S7.9 1-(*tert*-butyl) 2-methyl (2*S*,4*S*)-4-hydroxypyrrolidine-1,2-dicarboxylate 17

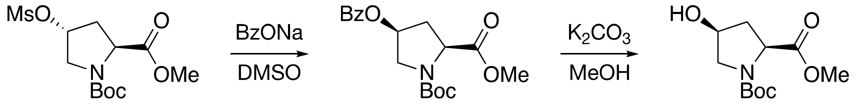

15 **20** **17**

In a round-bottom flask, 6.32 g mesyloxyproline **15** (19.5 mmol) was dissolved in 62 mL DMSO after which 5.64 g of sodium benzoate (39 mmol, 2 equiv.) was added. The reaction mixture was stirred at 90 °C for 17 h and cooled to 20 °C. The mixture was poured in 120 mL ethyl acetate and washed with 40 mL water and 50 mL brine. The organic phase was dried with $MgSO_4$ and the solvent was removed *in vacuo*. The resulting oil was crystallized in *n*-hexane, yielding 6.20 g of compound **20** as brownish crystals (91%).

Benzoyloxyproline **20** (6.20 g, 17.8 mmol) was dissolved in 120 mL methanol and 2.46 g K_2CO_3 (17.8 mmol, 1 equiv.) was added. The mixture was stirred during 1 h at 20 °C and diluted with 200 mL ethyl acetate. The organic layer was washed with brine, the aquatic phase was saturated with NaCl and extracted with chloroform. The chloroform extract was washed with brine and dried with $MgSO_4$. After removal of the solvent in vacuo, the resulting oil was purified with column chromatography ($KMnO_4$, *n*-hexane/EtOAc 1/3, R_f = 0.21) yielding 2.98 g of brownish crystals (68%). Spectra were in accordance with literature.[7]

S7.10 1-(*tert*-butyl) 2-methyl (2*S*,4*R*)-4-azidopyrrolidine-1,2-dicarboxylate 18

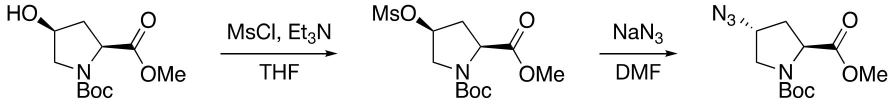

17 21 18

Analogous procedures were applied as were used for the synthesis of 1-(*tert*-butyl) 2-methyl (2*S*,4*R*)-4-((methylsulfonyl)oxy)pyrrolidine-1,2-dicarboxylate **15** and 1-(*tert*-butyl) 2-methyl (2*S*,4*S*)-4-azidopyrrolidine-1,2-dicarboxylate **16**.

S7.11 Methyl (2*S*,4*R*)-4-azidopyrrolidine-2-carboxylate hydrochloride 24

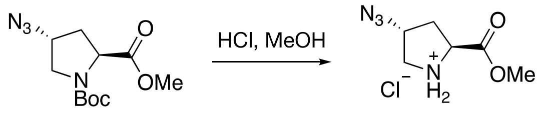

18 24

A round-bottom flask containing 100 mL of dry MeOH was cooled to 0 °C. Acetyl chloride (1.47 mL, 20.6 mmol, 4 equiv.) was added dropwise and the mixture was allowed to react for 20 minutes, after which azidoproline **18** (1.47 g, 5.4 mmol) was added. The solution was cooled to 0 °C and 1.55 mL acetyl chloride (2.18 mmol, 4 equiv.) was added. The reaction mixture was stirred for 1 h, after which the solvent and excess acid was removed evaporatively. The product was obtained as a light brown oil in a quantitative yield, used without further purification. Spectral data were in accordance with literature.[9]

S7.12 Methyl (2*S*,4*S*)-4-hydroxypyrrolidine-2-carboxylate hydrochloride 25

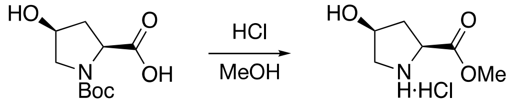

17 25

A round-bottom flask containing 100 mL dry MeOH was cooled to 0 °C. Acetyl chloride (1.47 mL, 20.6 mmol, 4 equiv.) was added dropwise and the mixture was allowed to react for 20 minutes, after which 1.26 g hydroxyproline **17** was added (5.14 mmol). The reaction mixture was stirred for 16 h, after which the solvent and excess acid was removed evaporatively. The product was obtained as a white solid in a quantitative yield, used without further purification. Spectral data in accordance with literature.[10]

S7.13 Typical procedure for the synthesis of diketopiperazines: cyclo(L-Pro-L-Pro) 1

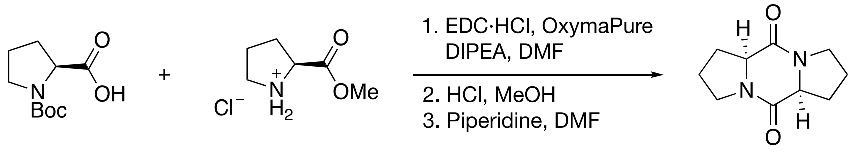

22 23 1

Proline **22** (3.0 g, 13.9 mmol) and methyl ester **23** (2.54 g, 15.3 mmol, 1.1 equiv.) were dissolved in 140 mL dry DMF under a nitrogen atmosphere. DIPEA (8.72 mL, 50.0 mmol, 3.6 equiv.), OxymaPure (2.37 g, 16.7 mmol, 1.2 equiv.) and EDC·HCl (3.2 g, 16.7 mmol, 1.2 equiv.) were added and the reaction mixture was stirred for 16 h at 20 °C. The organic solvent was removed *in vacuo* and the resulting dipeptide was obtained after reversed phase chromatography (water/ACN 95/5 to 75/25) as a yellow oil (3.86 g). The dipeptide was dissolved in 50 mL dry methanol under a nitrogen atmosphere to which 1.50 mL acetyl chloride (47.8 mmol, ~4 equiv.) was added dropwise at 0 °C. The reaction mixture was stirred for 16 h and the solvent was removed *in vacuo*, yielding a white foam. The foam was dissolved in 20 mL dry DMF under nitrogen atmosphere and 1.54 mL piperidine (36 mmol, ~3 equiv.) was added. The reaction mixture was stirred for 1 h and the solvent was removed *in vacuo*. Purification with reversed phase chromatography (water/ACN 100/0-20/80) yielded 0.90 g diketopiperazine (33%) as a white solid.[11]

This procedure was used to synthesize diketopiperazines **3-9**, Scheme S1 gives an overview of the proline analogs that were combined.

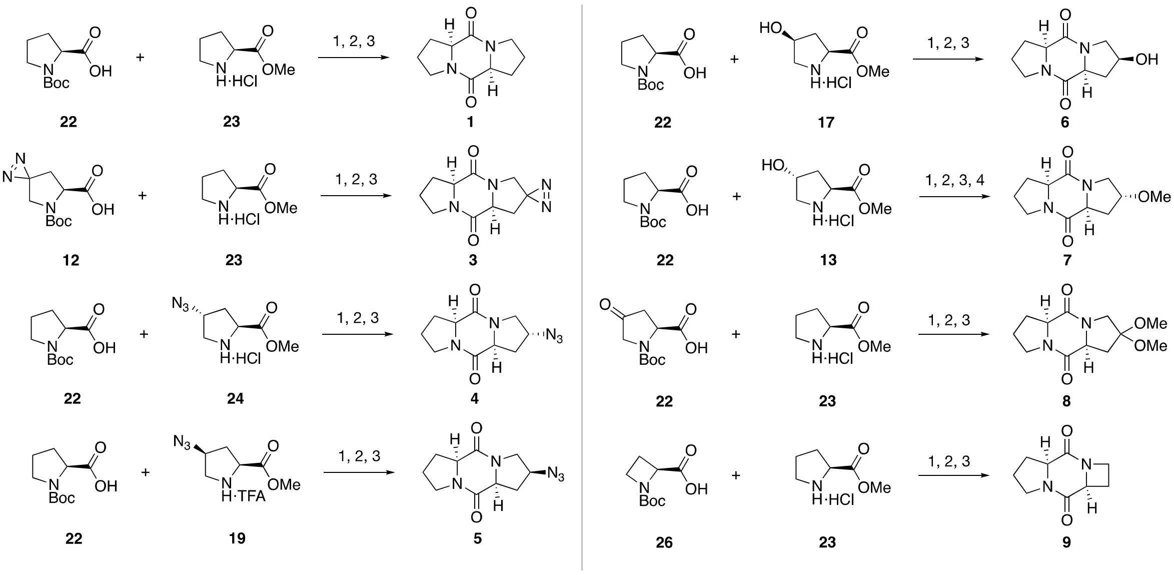

Scheme S1 | Synthesis of diketopiperazines 1, 3-9. Reagents and conditions: (1) EDC·HCl, OxymaPure, DIPEA, DMF, 16 h, 20 °C; (2) HCl, MeOH, 16 h, 0 °C to 20 °C; (3) piperidine, DMF, 1 h, 20 °C; (4) NaH, MeI, DMF, -10 °C to 20 °C; yield: 9 to 70% (for the yields of individual DKPs, see Section S8).

Diketopiperazine **8** was synthesized by coupling oxoproline **11** and H-Pro-OMe·HCl **23**. Acetal formation occurred during the acidic Boc-removal in methanol.

For diketopiperazine **7**, Boc-Pro-OH **22** was coupled with hydroxyproline **10** using the general procedure described above (yield: 19%). The resulting diketopiperazine (0.2 g, 0.95 mmol) was dissolved in 2 mL dry DMF. Methyl iodide (0.15 mL, 2.38 mmol, 2.5 equiv.) was added and the reaction mixture was cooled to -10 °C with an ice/NaCl bath. A 60% suspension of NaH in mineral oil (77 mg, 1.9 mmol, 2 equiv.) was added and the reaction mixture was stirred for 6 h. Ice water (5 mL) was added to the reaction mixture. The solvent was removed *in vacuo* and the resulting solid was purified using reversed phase chromatography (water/ACN 100/0-20/80), yielding 99 mg of a white solid (46%). The methylation procedure was adopted from Chiba *et al.*[12]

S8 NMR spectra of diketopiperazines.

S8.1 Diketopiperazine 1

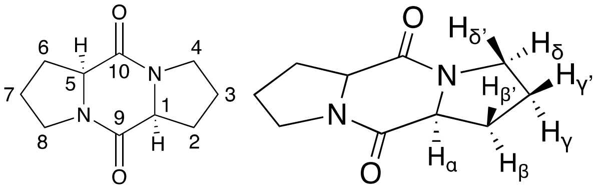

^{1}H NMR: 400 MHz, $CDCl_3$; ^{13}C NMR: 100.6 MHz, $CDCl_3$.

		**[1]H δ** [ppm]	**[13]C δ** [ppm]
1 and 5	α	4.18 (2H, t, J = 8.1 Hz)	60.6
2 and 6	β	2.33 (2H, d x t x d, J_1 = 13.5 Hz, J_2 = 6.7 Hz, J_3 = 3.5 Hz)	27.7
	β'	2.19 (2H, d x d x d x d, J_1 = 12.8 Hz, J_2 = 10.1 Hz, J_3 = 9.6 Hz, J_4 = 7.1 Hz)	
3 and 7	γ'	1.99–2.08 (2H, m)	23.4
	γ	1.92 (2H, d x d x t x d, J_1 = 12.7 Hz, J_2 = 10.6 Hz, J_3 = 8.6 Hz, J_4 = 6.8 Hz)	
4 and 8	δ	3.56 (2H, d x d x d, J_1 = 12.3 Hz, J_2 = 8.5 Hz, J_3 = 6.3 Hz)	45.2
	δ'	3.53 (2H, d x d x d, J_1 = 12.0 Hz, J_2 = 8.3 Hz, J_3 = 4.4 Hz)	
9 and 10		–	166.4

The assignment of β, β', γ, γ', δ and δ' is based on the work of Anteunis et al.,[13,14] Young et al.[15] and Haasnoot et al.[16]

^{1}H NMR

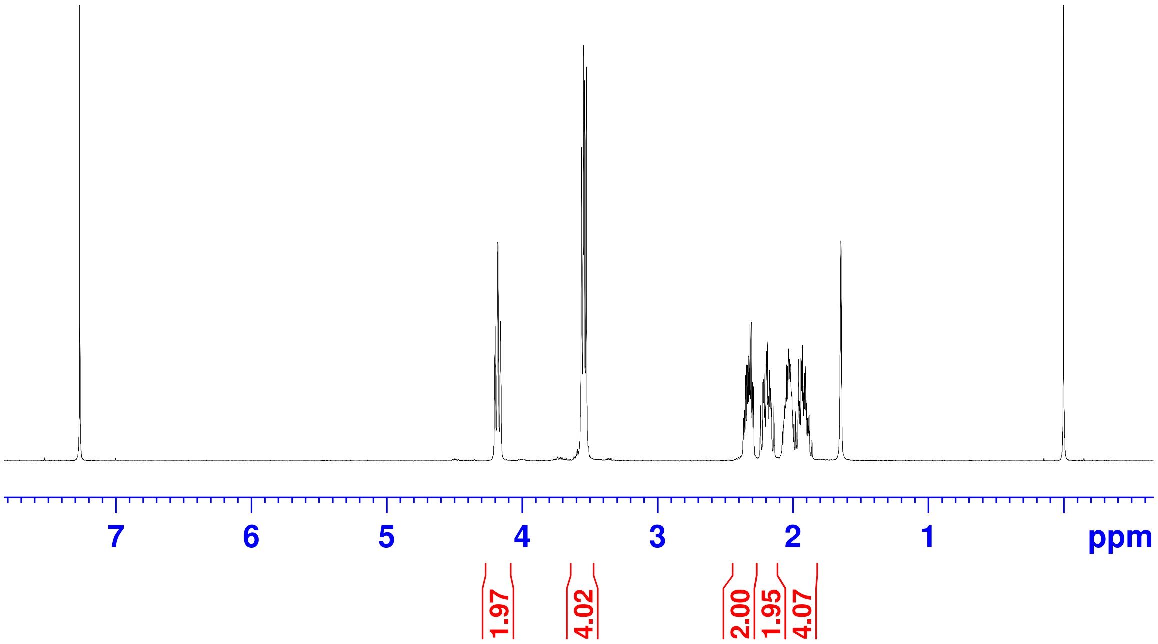

^{13}C NMR

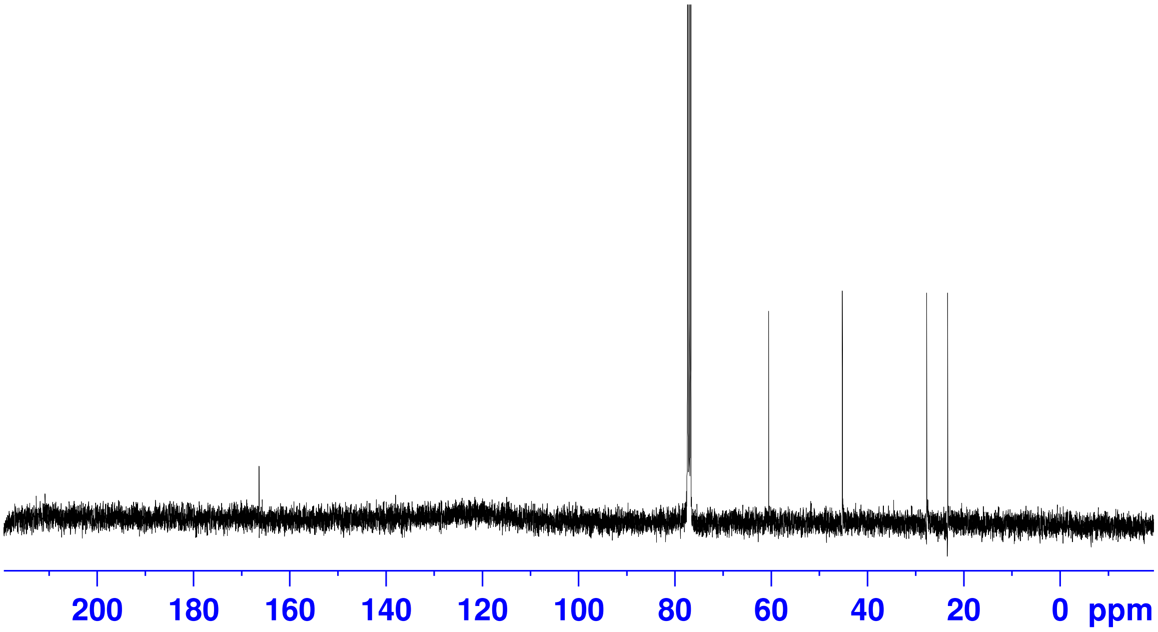

S8.2 Diketopiperazine 3

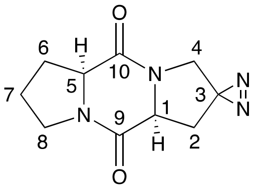

^{1}H NMR: 400 MHz, $CDCl_3$; ^{13}C NMR: 100.6 MHz, $CDCl_3$.

		Proline ring				Analogous ring	
		**^{1}H δ** [ppm]	**^{13}C δ** [ppm]			**^{1}H δ** [ppm]	**^{13}C δ** [ppm]
5	α	4.21 (1H, t,<br>J = 8.1 Hz)	60.0	1	α	4.63 (1H, t,<br>J = 8.7 Hz)	59.1
6	β	2.40 (1H, d x t x d,<br>J_1 = 13.0 Hz,<br>J_2 = 9.9 Hz,<br>J_3 = 3.0 Hz)	28.0	2	β and β’	2.53 (1H, d x d,<br>J_1 = 15.0 Hz,<br>J_2 = 9.3 Hz)	31.4
	β’	2.18 (1H, d x d x d x d,<br>J_1 = 12.9 Hz,<br>J_2 = 10.2 Hz,<br>J_3 = 9.9 Hz,<br>J_4 = 7.1 Hz)				1.74 (1H, d x d,<br>J_1 = 15.0 Hz,<br>J_2 = 8.4 Hz)	
7	γ’	2.07 (1H, d x t x t,<br>J_1 = 13.0 Hz,<br>J_2 = 6.6 Hz,<br>J_3 = 3.3 Hz)	23.1	3	γ’	–	29.7
	γ	1.95 (1H, d x d x t x d,<br>J_1 = 12.7 Hz,<br>J_2 = 10.8 Hz,<br>J_3 = 8.7 Hz,<br>J_4 = 6.8 Hz)			γ	–	
8	δ	3.61 (1H, d x d x d,<br>J_1 = 11.3 Hz,<br>J_2 = 9.5 Hz,<br>J_3 = 6.9 Hz)	45.6	4	δ and δ’	3.50 (1H, d,<br>J = 14.0 Hz)	47.4
	δ’	3.57 (1H, d x d x d,<br>J_1 = 11.5 Hz,<br>J_2 = 8.7 Hz,<br>J_3 = 3.5 Hz)				3.00 (1H, d,<br>J = 14.0 Hz)	
10		–	166.1	9		–	164.8

IR (FTIR, cm^{-1}): ν = 1667, 1431; HRMS (ESI): m/z calculated $C_{10}H_{13}N_2O_2$ $[M-N_2+H]^+$ 193.0972, found 193.0963; white solid; mp 168 °C; $[\alpha]_D^{27}$ -138.4 ± 3.9 (*c* 0.10, $CHCl_3$); yield: 79%.

^{1}H NMR

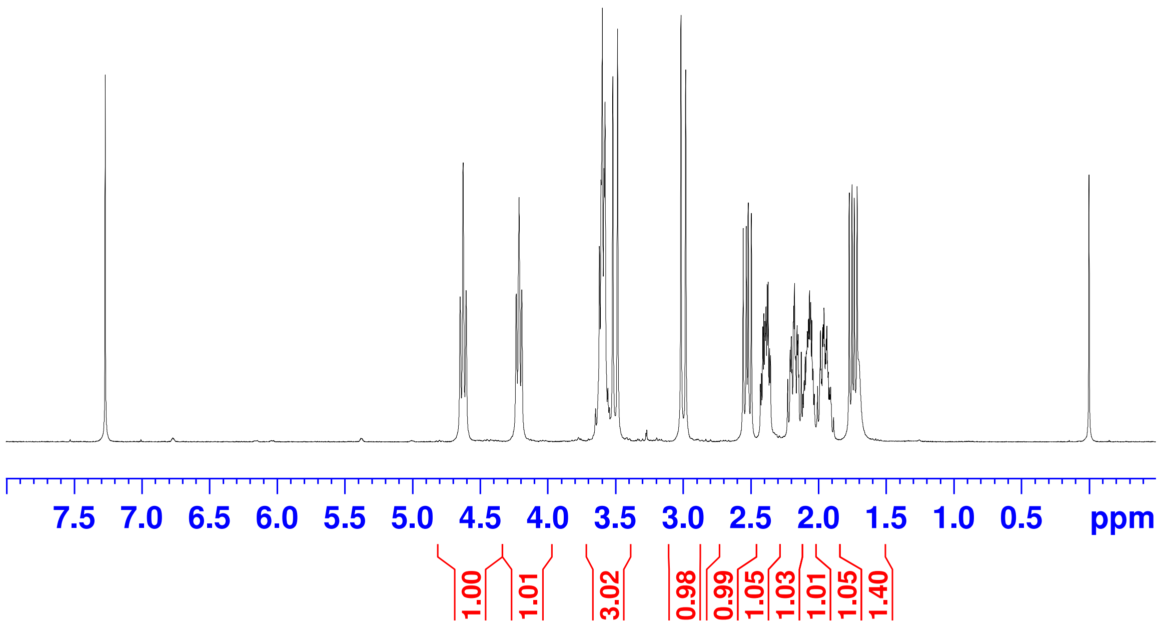

^{13}C NMR

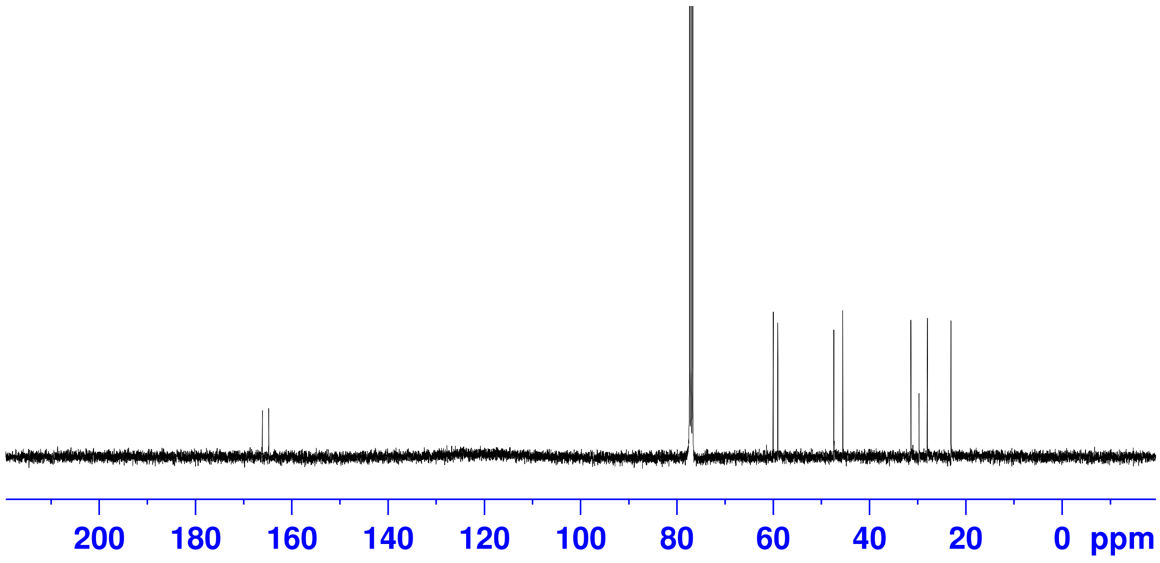

S8.3 Diketopiperazine 4

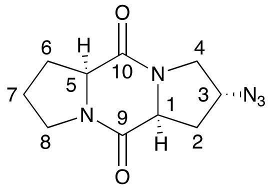

^{1}H NMR: 400 MHz, $CDCl_3$; ^{13}C NMR: 100.6 MHz, $CDCl_3$.

		Proline ring				Analogous ring	
		**^{1}H δ** [ppm]	**^{13}C δ** [ppm]			**^{1}H δ** [ppm]	**^{13}C δ** [ppm]
5	α	4.22 (1H, t, J = 8.1 Hz)	60.5	1	α	4.42 (1H, d x d, J_1 = 10.2 Hz, J_2 = 6.9 Hz)	58.8
6	β<br>β'	2.34 (1H, d x t x d, J_1 = 13.6 Hz, J_2 = 6.8 Hz, J_3 = 3.3 Hz)<br>2.19 (1H, d x d x d x d, J_1 = 12.7 Hz, J_2 = 10.4 Hz, J_3 = 9.4 Hz, J_4 = 6.9 Hz)	27.6	2	β<br>β'	2.44 (1H, d x d x t, J_1 = 13.8 Hz, J_2 = 6.6 Hz, J_3 = 1.4 Hz)<br>2.32 (1H, d x d x d, J_1 = 13.9 Hz, J_2 = 10.4 Hz, J_3 = 4.7 Hz)	34.0
7	γ'<br>γ	2.04 (1H, d x d x t x d, J_1 = 12.7 Hz, J_2 = 7.2 Hz, J_3 = 5.6 Hz, J_4 = 3.6 Hz)<br>1.94 (1H, d x d x t x d, J_1 = 12.6 Hz, J_2 = 10.5 Hz, J_3 = 8.5 Hz, J_4 = 6.8 Hz)	23.4	3	γ'<br>γ	4.35 (1H, t x t, J_1 = 5.0 Hz, J_2 = 1.4 Hz)<br>–	58.7
8	δ and δ'	3.55 (2H, d x d, J_1 = 8.5 Hz, J_2 = 5.5 Hz)	45.3	4	δ'<br>δ	3.72 (1H, d x d, J_1 = 13.0 Hz, J_2 = 5.2 Hz)<br>3.62 (1H, d x t, J_1 = 13.0 Hz, J_2 = 1.35 Hz)	50.7
10		–	166.3	9			165.7

IR (FTIR, cm^{-1}): ν = 2099, 1657, 1431; HRMS (ESI): m/z calculated $C_{10}H_{14}N_5O_2$ $[M+H]^+$ 236.1142, found 236.1150; yellowish crystals, after preparative HPLC: white crystals; mp 168 °C; $[\alpha]_D^{25}$ -144.3 ± 3.8 (*c* 0.12, $CHCl_3$); yield: 78%.

^{1}H NMR

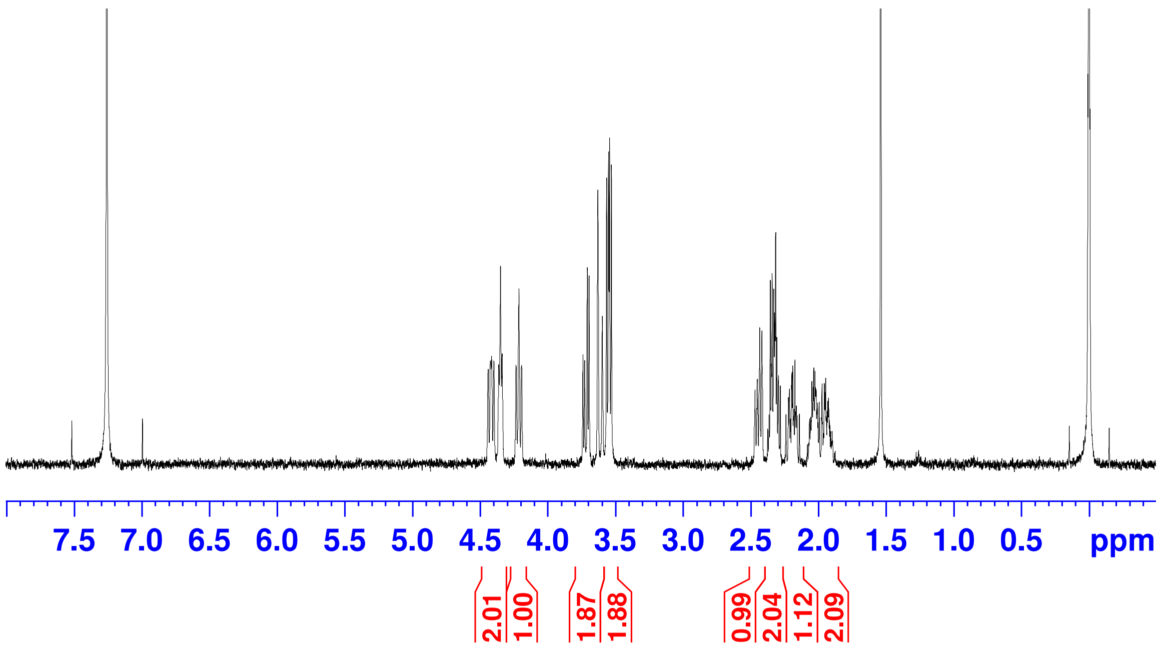

^{13}C NMR

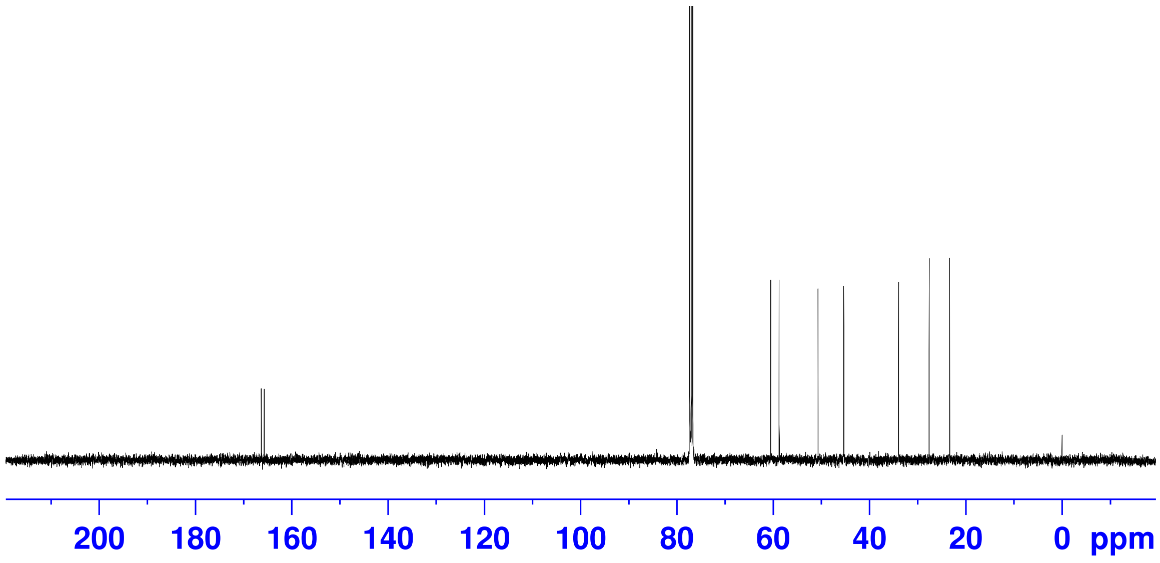

S8.4 Diketopiperazine 5

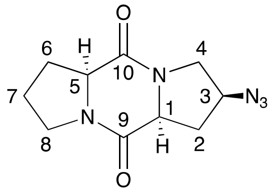

^{1}H NMR: 400 MHz, $CDCl_3$; ^{13}C NMR: 100.6 MHz, $CDCl_3$.

		Proline ring				Analogous ring	
		**^{1}H δ** [ppm]	**^{13}C δ** [ppm]			**^{1}H δ** [ppm]	**^{13}C δ** [ppm]
5	α	Overlap with proton #3<br>4.14–4.21 (2H, m)	60.2	1	α	4.27 (1H, d x d,<br>J_1 = 7.4 Hz)	58.7
6	β<br><br><br><br>β’	2.33 (1H, d x t x d,<br>J_1 = 13.3 Hz,<br>J_2 = 6.9 Hz,<br>J_3 = 3.8 Hz)<br>2.24 (1H, d x d x d x d,<br>J_1 = 13.0 Hz,<br>J_2 = 9.9 Hz,<br>J_3 = 9.5 Hz,<br>J_4 = 7.2 Hz)	27.6	2	β<br><br><br><br>β’	2.67 (1H, d x t,<br>J_1 = 13.7 Hz,<br>J_2 = 5.6 Hz)<br><br>2.52 (1H, d x d x d,<br>J_1 = 14.0 Hz,<br>J_2 = 8.5 Hz,<br>J_3 = 5.6 Hz)	32.8
7	γ’<br><br><br><br>γ	2.05 (1H, d x t x t,<br>J_1 = 13.5 Hz,<br>J_2 = 6.8 Hz,<br>J_3 = 3.5 Hz)<br>1.91 (1H, d x d x t x d,<br>J_1 = 12.7 Hz,<br>J_2 = 10.1 Hz,<br>J_3 = 8.6 Hz,<br>J_4 = 7.1 Hz)	23.3	3	γ	Overlap with proton #5<br>4.14–4.21 (2H, m)	58.2
8	δ and δ’	Both protons overlap with proton #4<br>3.51–3.63 (3H, m)	45.5	4	δ and δ’	3.78 (1H, d x d,<br>J_1 = 12.4 Hz,<br>J_2 = 3.9 Hz)<br>One proton overlaps with protons at #8<br>3.51–3.63 (3H, m)	50.3
10		–	166.8	9		–	165.2

IR (FTIR, cm^{-1}): ν = 2099, 1657, 1420; HRMS (ESI): *m/z* calculated $C_{10}H_{14}N_5O_2$ $[M+H]^+$ 236.1142, found 236.1131; yellowish oil, colorless oil after preparative HPLC; $[\alpha]_D^{27}$ -19.8 ± 4.8 (*c* 0.12, $CHCl_3$); yield: 72%.

^{1}H NMR

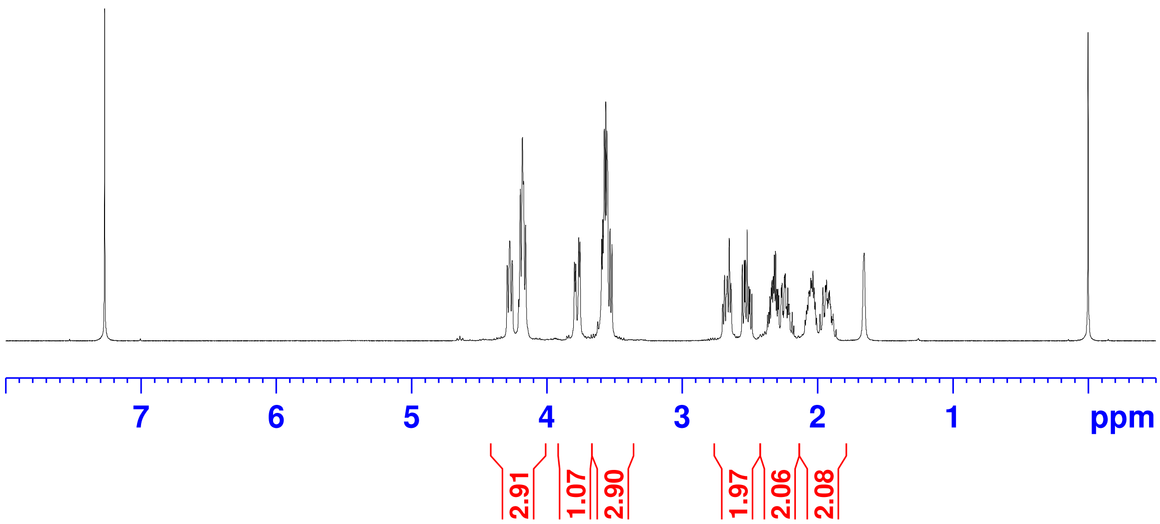

^{13}C NMR

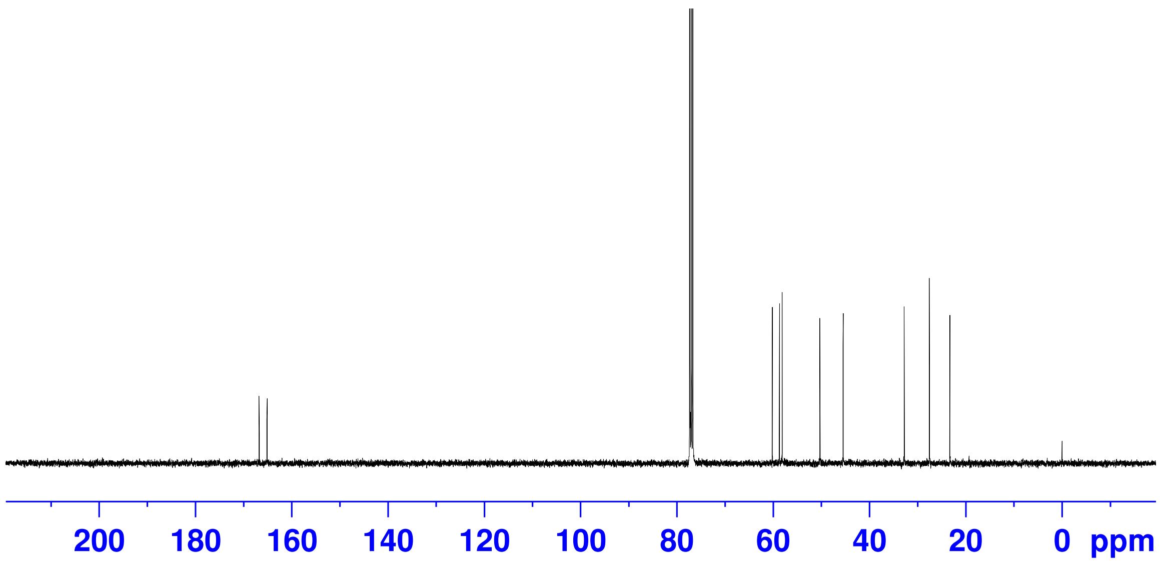

S8.5 Diketopiperazine 6

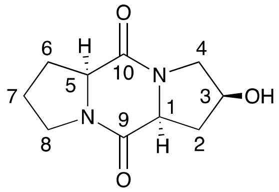

^{1}H NMR: 400 MHz, $CDCl_3$; ^{13}C NMR: 100.6 MHz, $CDCl_3$.

		Proline ring				Analogous ring	
		**^{1}H δ** [ppm]	**^{13}C δ** [ppm]			**^{1}H δ** [ppm]	**^{13}C δ** [ppm]
5	α	4.19 (1H, t,<br>J = 8.16 Hz)	60.3	1	α	4.25 (1H, d x d,<br>J_1 = 9.18 Hz,<br>J_2 = 4.98 Hz)	58.8
6	β<br><br><br><br><br>β’	2.33 (1H, d x t x d,<br>J_1 = 13.1 Hz,<br>J_2 = 7.3 Hz,<br>J_3 = 3.9 Hz)<br><br>2.25 (1H, d x d x d x d,<br>J_1 = 13.0 Hz,<br>J_2 = 10.1 Hz,<br>J_3 = 8.9 Hz,<br>J_4 = 7.0 Hz)	27.5	2	β<br><br><br><br><br>β’	2.62 (1H, d x d x d x d,<br>J_1 = 13.9 Hz,<br>J_2 = 4.9 Hz,<br>J_3 = 3.6 Hz,<br>J_4 = 1.5 Hz)<br>2.41 (1H, d x d x d,<br>J_1 = 13.9 Hz,<br>J_2 = 9.2 Hz,<br>J_3 = 4.8 Hz)	35.6
7	γ’<br><br><br><br><br><br>γ	2.04 (1H, d x d x d x d,<br>J_1 = 10.7 Hz,<br>J_2 = 8.0 Hz,<br>J_3 = 6.1 Hz,<br>J_4 = 4.7 Hz,<br>J_5 = 4.6 Hz)<br>1.92 (1H, d x d x t x d,<br>J_1 = 12.6 Hz,<br>J_2 = 10.3 Hz,<br>J_3 = 8.6 Hz,<br>J_4 = 7.0 Hz)	23.4	3	γ	4.52 (1H, t x t,<br>J_1 = 4.6 Hz,<br>J_2 = 3.2 Hz)	69.1
8	δ<br><br><br><br>δ’	3.57 (1H, d x d x d,<br>J_1 = 12.6 Hz,<br>J_2 = 7.8 Hz,<br>J_3 = 5.3 Hz)<br>3.54 (1H, d x d x d,<br>J_1 = 11.9 Hz,<br>J_2 = 8.1 Hz,<br>J_3 = 4.5 Hz)	45.4	4	δ and δ’	3.81 (1H, d x t,<br>J_1 = 12.2 Hz,<br>J_2 = 2.1 Hz)<br><br>3.41 (1H, d x d,<br>J_1 = 12.3 Hz,<br>J_2 = 4.3 Hz)	53.2
10		–	167.3	9		–	166.3

IR (ATR, cm^{-1}): ν = 3397, 2108, 1643; HRMS (ESI): *m/z* calculated $C_{10}H_{15}N_2O_3$ $[M+H]^+$ 211.1077, found 211.1072; white crystals; mp 160.6 °C; $[\alpha]_D^{24}$ -128.1 ± 5.2 (*c* 0.19, $CHCl_3$); yield: 30%.

^{1}H NMR

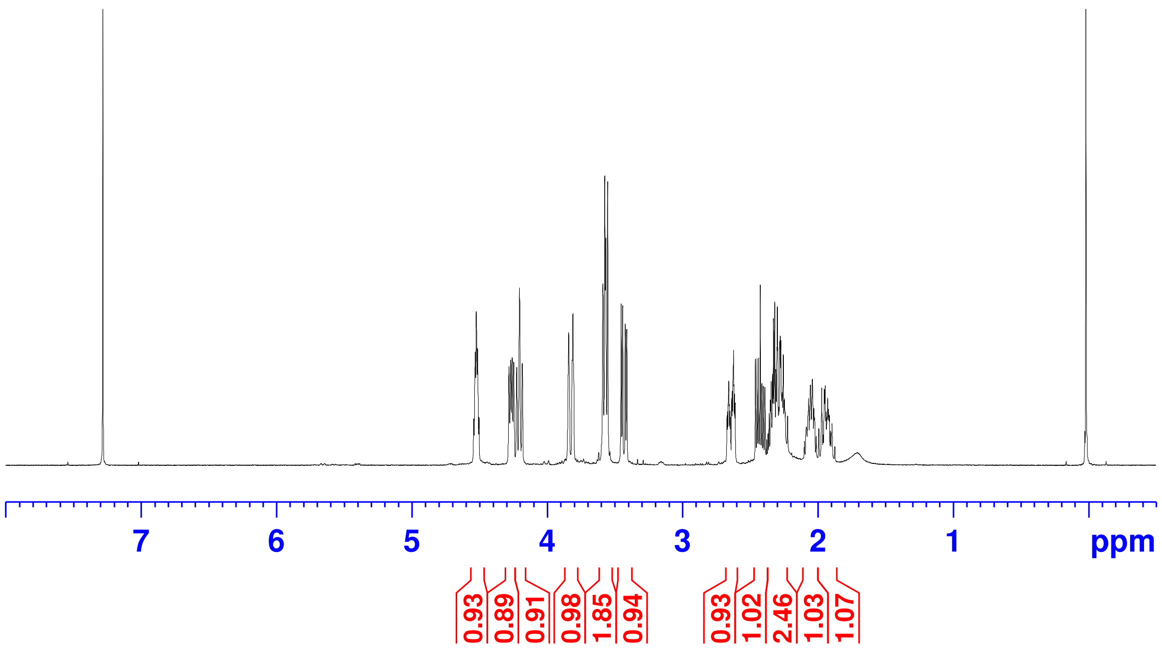

^{13}C NMR

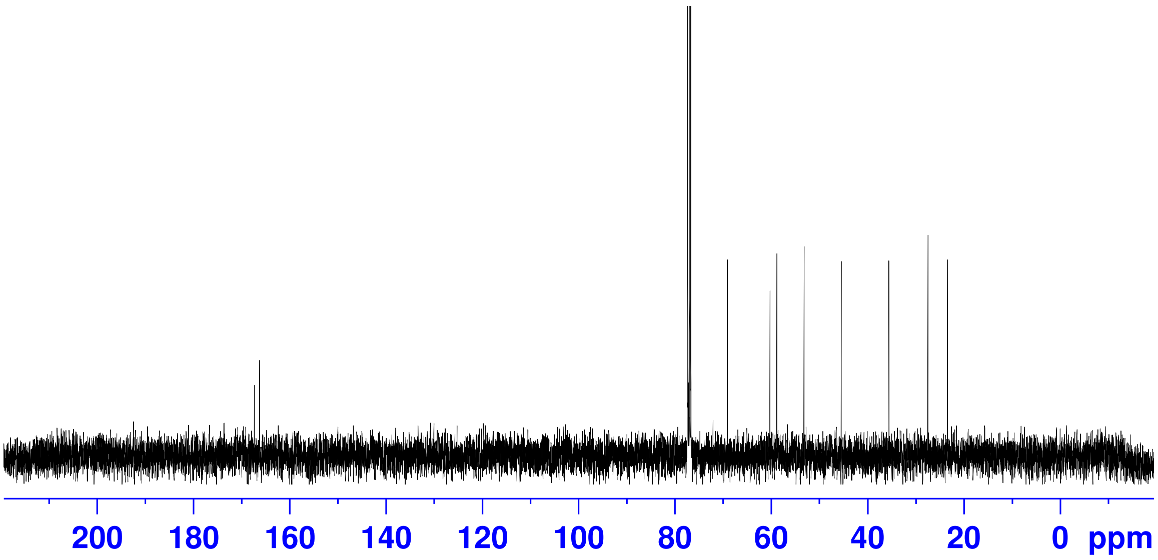

S8.6 Diketopiperazine 7

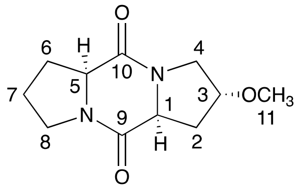

^{1}H NMR: 400 MHz, $CDCl_3$; ^{13}C NMR: 100.6 MHz, $CDCl_3$.

		Proline ring				Analogous ring	
		**^{1}H δ** [ppm]	**^{13}C δ** [ppm]			**^{1}H δ** [ppm]	**^{13}C δ** [ppm]
5	α	4.19 (1H, t, J = 8.2 Hz)	60.5	1	α	4.41 (1H, d x d, J_1 = 10.5 Hz, J_2 = 6.7 Hz)	58.9
6	β<br>β’	2.33 (1H, d x t x d, J_1 = 13.8 Hz, J_2 = 6.6 Hz, J_3 = 3.4 Hz)<br>2.18 (1H, d x d x d x d, J_1 = 12.9 Hz, J_2 = 10.6 Hz, J_3 = 9.2 Hz, J_4 = 7.2 Hz)	27.7	2	β<br>β’	2.51 (1H, d x d x t, J_1 = 13.8 Hz, J_2 = 6.6 Hz, J_3 = 1.4 Hz)<br>2.11 (1H, d x d x d, J_1 = 13.8 Hz, J_2 = 10.9 Hz, J_3 = 4.4 Hz)	33.6
7	γ’<br>γ	2.03 (1H, d x d x t x d, J_1 = 12.7 Hz, J_2 = 7.2 Hz, J_3 = 5.5 Hz, J_4 = 3.5 Hz)<br>1.92 (1H, d x d x t x d, J_1 = 12.7 Hz, J_2 = 10.7 Hz, J_3 = 8.6 Hz, J_4 = 6.7 Hz)	23.3	3	γ’<br>γ	4.04 (1H, t, J_1 = 4.3 Hz)<br>–	77.9
8	δ and δ’	3.54 (2H, d x d, J_1 = 8.6 Hz, J_2 = 5.5 Hz)	45.3	4	δ’<br>δ	3.66 (1H, br. d, J = 13.1 Hz)<br>3.59 (1H, d x d, J_1 = 13.1 Hz, J_2 = 4.3 Hz)	50.6
9 and 10		–	166.4<br>166.5	11		3.37 (3H, s)	56.5

IR (FTIR, cm^{-1}): ν = 1643, 1431; HRMS (ESI): *m/z* calculated $C_{11}H_{17}N_2O_3$ $[M+H]^+$ 225.1234, found 225.1239; white solid; mp 160 °C; $[\alpha]_D^{26}$ -130.6 ± 4.0 (*c* 0.11, $CHCl_3$); yield: 9% (see page 21 for details).

^{1}H NMR

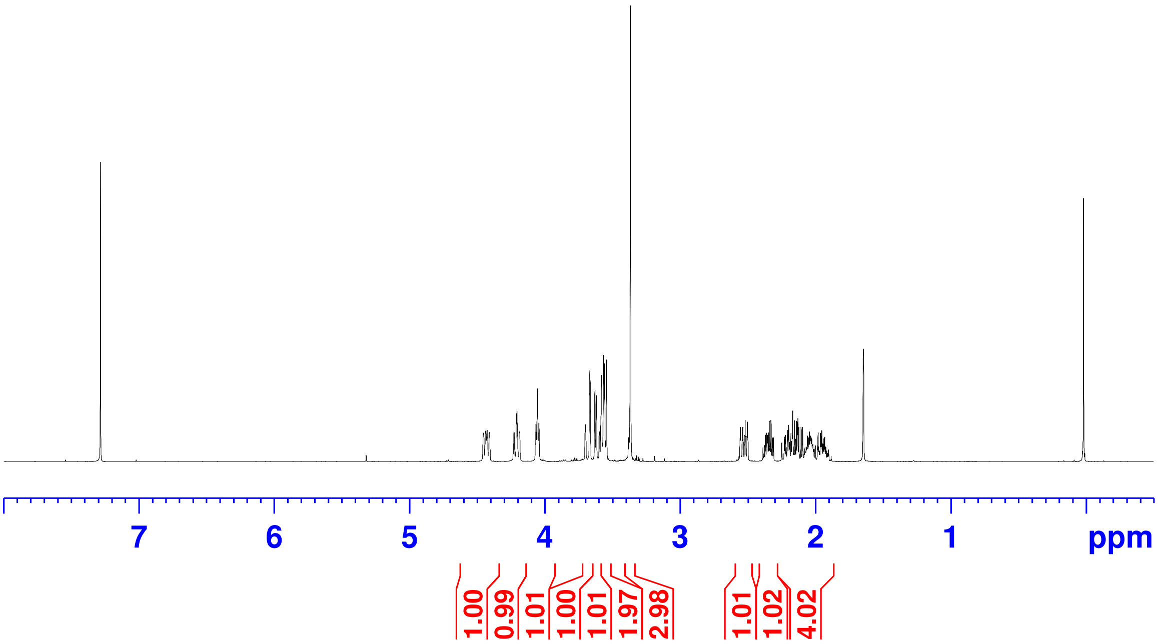

^{13}C NMR (APT)

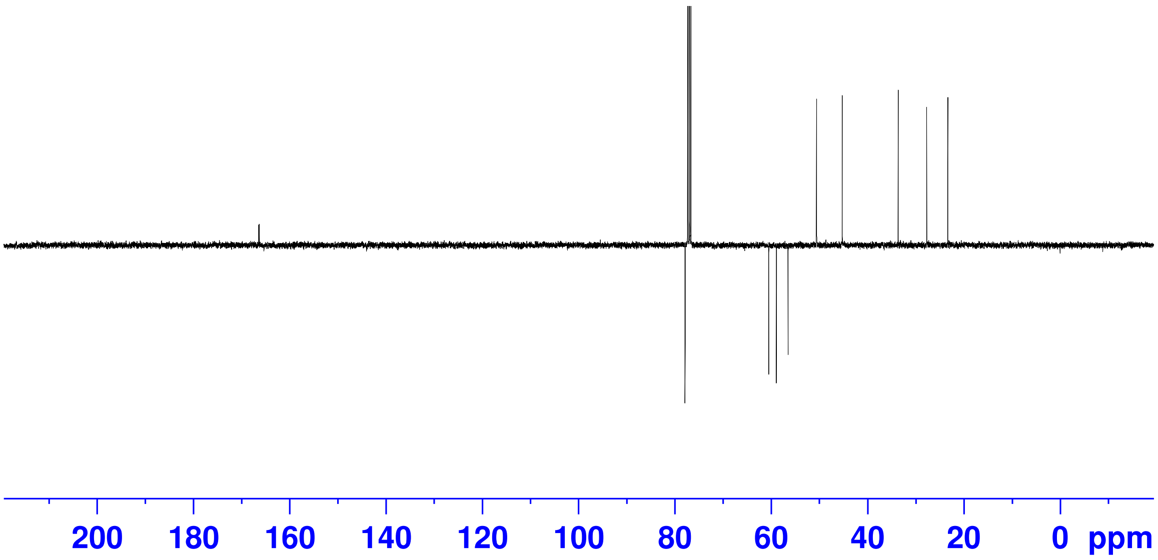

S8.7 Diketopiperazine 8

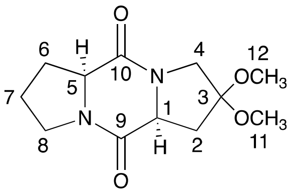

^{1}H NMR: 400 MHz, $CDCl_3$; ^{13}C NMR: 100.6 MHz, $CDCl_3$.

		Proline ring				Analogous ring	
		**^{1}H δ** [ppm]	**^{13}C δ** [ppm]			**^{1}H δ** [ppm]	**^{13}C δ** [ppm]
5	α	4.14 (1H, t, J = 8.2 Hz)	60.0	1	α	4.34 (1H, t, J = 8.5 Hz)	59.0
6	β<br>β’	2.36 (1H, d x t x d, J_1 = 13.0 Hz, J_2 = 6.8 Hz, J_3 = 3.4 Hz)<br>2.20 (1H, d x d x d x d, J_1 = 13.0 Hz, J_2 = 10.6 Hz, J_3 = 9.3 Hz, J_4 = 7.2 Hz)	27.7	2	β<br>β’	2.52 (1H, d x d x d, J_1 = 13.2 Hz, J_2 = 7.5 Hz, J_3 = 1.2 Hz)<br>2.36 (1H, d x d x d, J_1 = 13.1 Hz, J_2 = 9.6 Hz, J_3 = 0.6 Hz)	35.3
7	γ’<br>γ	1.98–2.07 (1H, m)<br>1.91 (1H, d x d x t x d, J_1 = 12.8 Hz, J_2 = 10.7 Hz, J_3 = 8.7 Hz, J_4 = 6.7 Hz)	23.3	3	γ’<br>γ	–<br>–	106.2
8	δ<br>δ’	3.57 (1H, d x d x d, J_1 = 11.7 Hz, J_2 = 8.7 Hz, J_3 = 6.9 Hz)<br>3.54 (1H, d x d x d, J_1 = 11.8 Hz, J_2 = 8.0 Hz, J_3 = 4.4 Hz)	45.4	4	δ and δ’	3.68 (1H, d, J = 12.2 Hz)<br>3.57 (1H, d, J = 11.9 Hz)	51.3
9<br>10		–	165.8<br>166.6	11 + 12		3.27 (3H, s)<br>3.27 (3H, s)	49.3<br>50.6

IR (FTIR, cm^{-1}): ν =1661, 1429; HRMS (ESI): *m/z* calculated $C_{11}H_{15}N_2O_3$ $[M-MeOH+H]^+$ 223.1077, found: 223.1070; dark brownish oil, yellowish crystals after preparative HPLC; mp 195 °C; R_f = 0.27 (ACN/*i*PrOH 90/10); $[\alpha]_D^{28}$ -104.8 ± 2.1 (*c* 0.13, $CHCl_3$); yield: 29%.

^{1}H NMR

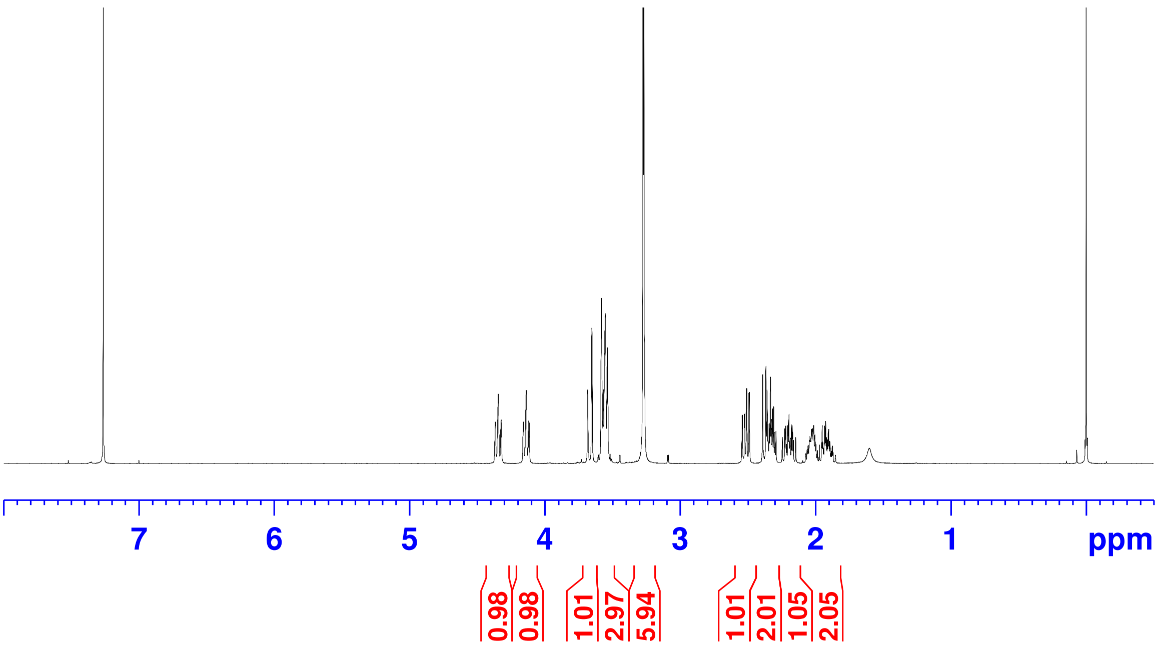

^{13}C NMR

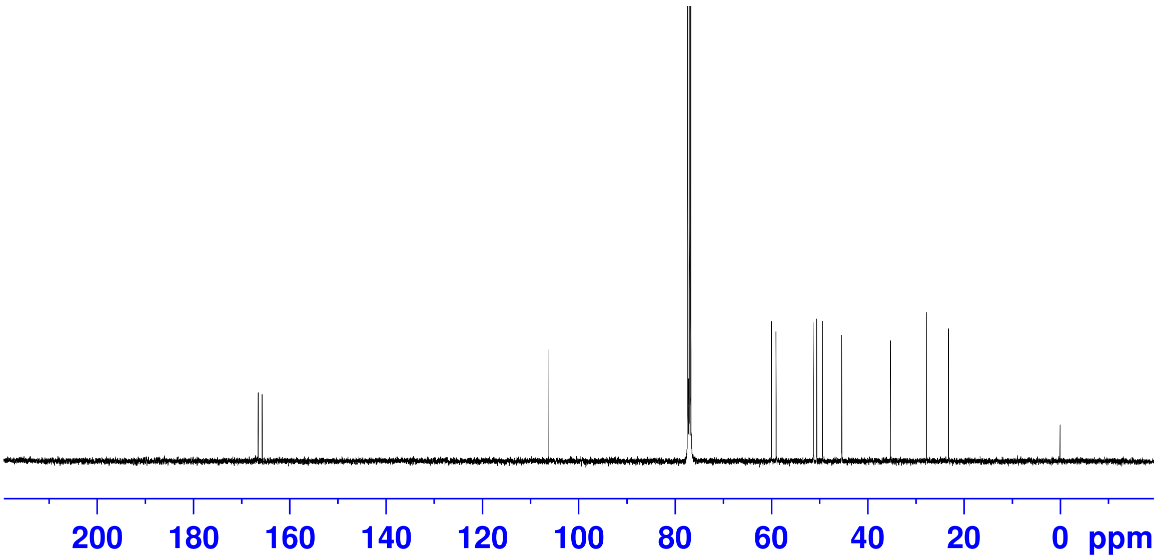

S8.8 Diketopiperazine 9

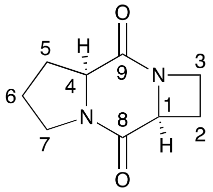

^{1}H NMR: 400 MHz, $CDCl_3$; ^{13}C NMR: 100.6 MHz, $CDCl_3$.

		Proline ring				Analogous ring	
		**^{1}H δ** [ppm]	**^{13}C δ** [ppm]			**^{1}H δ** [ppm]	**^{13}C δ** [ppm]
4	α	4.07 (1H, t, J = 9.2 Hz)	59.0	1	α	4.93 (1H, t, J = 7.8 Hz)	63.0
5	β<br>β'	2.28 (1H, d x d x d x d, J_1 = 13.0 Hz, J_2 = 10.0 Hz, J_3 = 8.7 Hz, J_4 = 7.0 Hz)<br>2.22 (1H, d x d x d x d, J_1 = 13.4 Hz, J_2 = 7.0 Hz, J_3 = 6.9 Hz, J_4 = 4.5 Hz)	26.9	2	β<br>β'	2.69 (1H, t x t, J_1 = 11.7 Hz, J_2 = 7.5 Hz)<br>2.66 (1H, d x d x d x d, J_1 = 12.0 Hz, J_2 = 11.6 Hz, J_3 = 8.1 Hz, J_4 = 7.2 Hz)	22.7
6	γ'<br>γ	2.01 (1H, d x d x d x t, J_1 = 11.5 Hz, J_2 = 8.4 Hz, J_3 = 7.2 Hz, J_4 = 4.3 Hz)<br>1.91 (1H, d x d x d x d x d, J_1 = 12.7 Hz, J_2 = 10.1 Hz, J_3 = 8.5 Hz, J_4 = 8.3 Hz, J_5 = 6.8 Hz)	23.2	3	γ and γ'	4.06 (1H, d x t, J_1 = 9.0 Hz, J_2 = 8.0 Hz)<br>4.04 (1H, q, J = 9.5 Hz)	47.6
7	δ<br>δ'	3.60 (1H, d x t, J_1 = 11.8 Hz, J_2 = 8.0 Hz)<br>3.53 (1H, d x d x d, J_1 = 12.0 Hz, J_2 = 8.2 Hz, J_3 = 4.1 Hz)	45.6				
9		–	169.0	8		–	167.3

IR (ATR, cm^{-1}): ν = 1667, 1449; HRMS (ESI): *m/z* calculated $C_9H_{13}N_2O_2$ $[M+H]^+$ 181.0972, found 181.0975; white crystals; mp 174 °C (lit.[17] mp 131–133 °C); R_f = 0.28 (ACN/*i*PrOH 90/10); $[\alpha]_D^{27}$ -77.0 ± 2.3 (*c* 0.12, $CHCl_3$); yield 62%.

^{1}H NMR

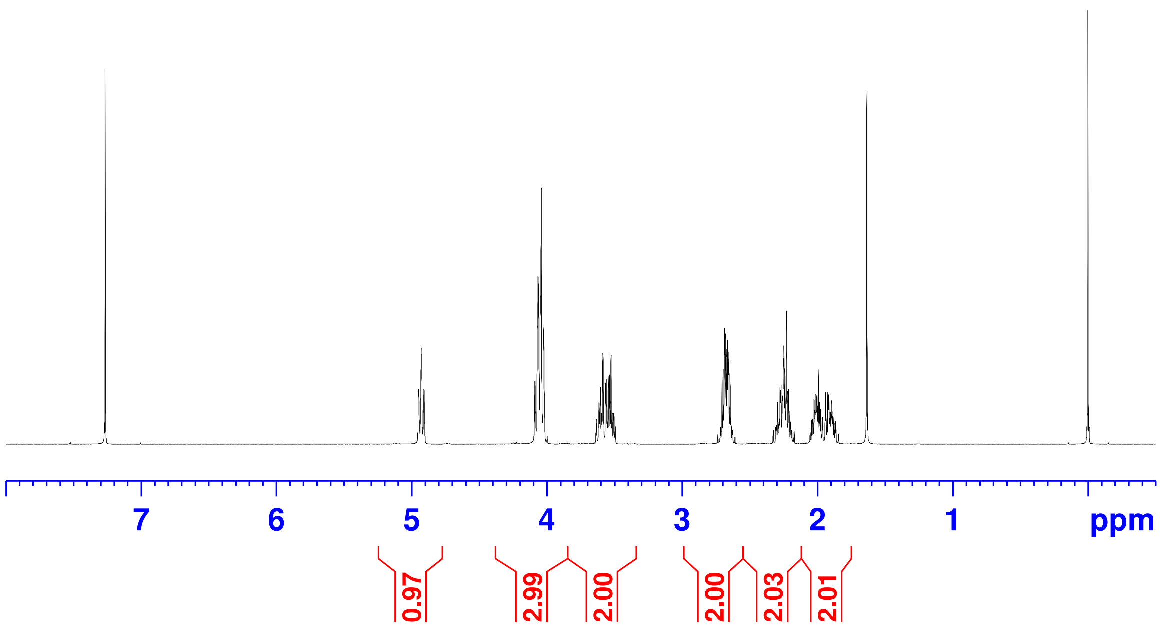

^{13}C NMR

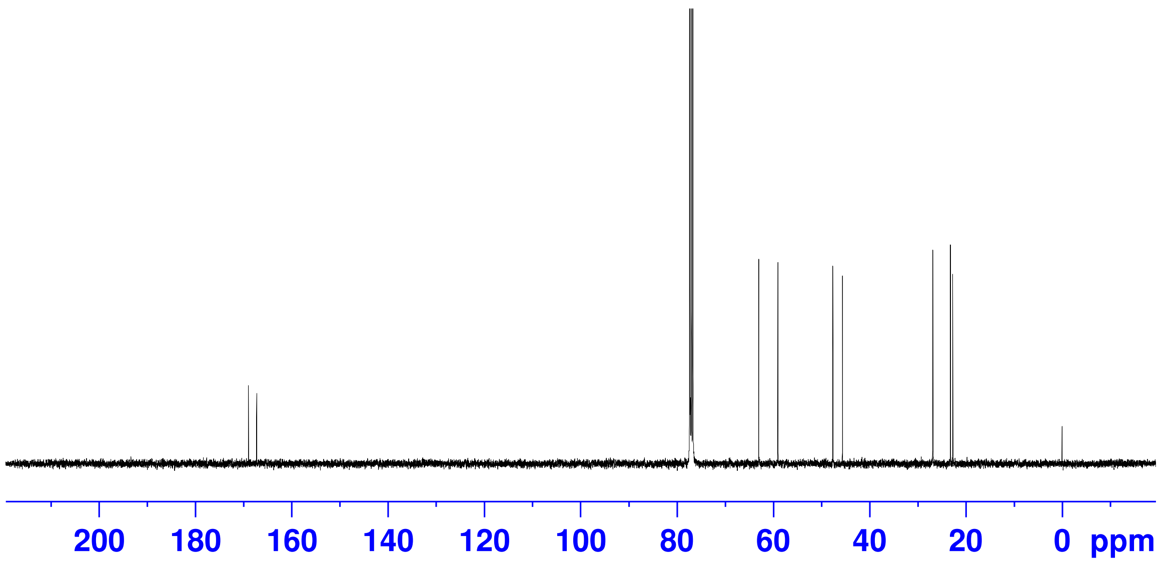

References

(1) Siemion, I. Z.; Wieland, T.; Pook, K.-H. Influence of the Distance of the Proline Carbonyl from the β and γ Carbon on the 13C Chemical Shifts. *Angew. Chemie Int. Ed. English* **1975**, *14* (10), 702–703. https://doi.org/10.1002/anie.197507021.

(2) Benedetti, E.; Goodman, M.; Marsh, R.; Rapoport, H.; Musich, J. Cyclo-L-Prolyl-L-Prolyl, C10H14N2O2. *Cryst. Struct. Commun.* **1975**, *4* (4), 641–645.

(3) Keller, O.; Keller, W. E.; van Look, G.; Wersin, G. Tert-Butoxycarbonylation of Amino Acids and Their Derivatives: N-Tert-Butoxycarbonyl-l-Phenylalanine. *Org. Synth.* **1985**, *63*, 160–170. https://doi.org/10.15227/orgsyn.063.0160.

(4) Van der Meijden, B.; Robinson, J. A. Synthesis and Application of Photoproline - a Photoactivatable Derivative of Proline. *Arkivoc* **2011**, *4*, 130–136. https://doi.org/10.3998/ark.5550190.0012.611.

(5) Chalker, J. M.; Gunnoo, S. B.; Boutureira, O.; Gerstberger, S. C.; Fernández-González, M.; Bernardes, G. J. L.; Griffin, L.; Hailu, H.; Schofield, C. J.; Davis, B. G. Methods for Converting Cysteine to Dehydroalanine on Peptides and Proteins. *Chem. Sci.* **2011**, *2* (9), 1666. https://doi.org/10.1039/c1sc00185j.

(6) Ji, X.; Xia, C.; Wang, J.; Su, M.; Zhang, L.; Dong, T.; Li, Z.; Wan, X.; Li, J.; Li, J.; et al. Design, Synthesis and Biological Evaluation of 4-Fluoropyrrolidine-2-Carbonitrile and Octahydrocyclopenta[b]Pyrrole-2-Carbonitrile Derivatives as Dipeptidyl Peptidase IV Inhibitors. *Eur. J. Med. Chem.* **2014**, *86*, 242–256. https://doi.org/10.1016/j.ejmech.2014.08.059.

(7) Marusawa, H.; Setoi, H.; Sawada, A.; Kuroda, A.; Seki, J.; Motoyama, Y.; Tanaka, H. Synthesis and Biological Activity of 1-Phenylsulfonyl-4-Phenylsulfonylaminopyrrolidine Derivatives as Thromboxane A2 Receptor Antagonists. *Bioorg. Med. Chem.* **2002**, *10* (5), 1399–1415. https://doi.org/10.1016/S0968-0896(01)00397-2.

(8) Abraham, D. J.; Mokotoff, M.; Sheh, L.; Simmons, J. E. Design, Synthesis, and Testing of Antisickling Agents. 2. Proline Derivatives Designed for the Donor Site. *J. Med. Chem.* **1983**, *26* (4), 549–554. https://doi.org/10.1021/jm00358a017.

(9) Chen, A. C.-Y.; Gomez, R.; Oballa, R. M.; Powell, D. A.; Roppe, J. R.; Seiders, T. J.; Sheng, T. Preparation of Aliphatic Prolinamide Derivatives as HTRA1 Serine Protease Inhibitors., 28 December 2017.

(10) Wood, J. M.; Furkert, D. P.; Brimble, M. A. Total Synthesis and Stereochemical

Revision of the 2-Formylpyrrole Alkaloid Hemerocallisamine I. *J. Nat. Prod.* **2017**, *80* (6), 1926–1929. https://doi.org/10.1021/acs.jnatprod.7b00314.

(11) Campbell, J.; Lin, Q.; Geske, G. D.; Blackwell, H. E. New and Unexpected Insights into the Modulation of LuxR-Type Quorum Sensing by Cyclic Dipeptides. *ACS Chem. Biol.* **2009**, *4* (12), 1051–1059. https://doi.org/10.1021/cb900165y.

(12) Chiba, J.; Takayama, G.; Takashi, T.; Yokoyama, M.; Nakayama, A.; Baldwin, J. J.; McDonald, E.; Moriarty, K. J.; Sarko, C. R.; Saionz, K. W.; et al. Synthesis, Biological Evaluation, and Pharmacokinetic Study of Prolyl-1-Piperazinylacetic Acid and Prolyl-4-Piperidinylacetic Acid Derivatives as VLA-4 Antagonists. *Bioorg. Med. Chem.* **2006**, *14* (8), 2725–2746. https://doi.org/10.1016/j.bmc.2005.11.058.

(13) Anteunis, M. J. O.; Callens, R.; Asher, V.; Sleeckx, J. Ring Conformational Aspects of Proline and Hydroxyproline. High Conformational Purity of Pro in DKP's and Hydantoïns. *Bull. des Sociétés Chim. Belges* **2010**, *87* (1), 41–60. https://doi.org/10.1002/bscb.19780870106.

(14) Sleeckx, J. J. M.; Anteunis, M. J. O. Revisited Conformational Aspects of Pro and Hyp Pyrrolidine Rings In Bicyclic Peptide Systems. *Bull. des Sociétés Chim. Belges* **2010**, *94* (3), 187–198. https://doi.org/10.1002/bscb.19850940306.

(15) Young, P. E.; Madison, V.; Blout, E. R. Cyclic Peptides. VI. Europium-Assisted Nuclear Magnetic Resonance Study of the Solution Conformations of Cyclo(L-Pro-L-Pro) and Cyclo(L-Pro-D-Pro). *J. Am. Chem. Soc.* **1973**, *95* (18), 6142–6144. https://doi.org/10.1021/ja00799a065.

(16) Haasnoot, C. A. G.; de Leeuw, F. A. A. M.; Altona, C. The Relationship between Proton-Proton NMR Coupling Constants and Substituent Electronegativities – I. *Tetrahedron* **1980**, *36* (19), 2783–2792. https://doi.org/10.1016/0040-4020(80)80155-4.

(17) Vičar, J.; Smolíková, J.; Bláha, K. Amino Acids and Peptides. CXV. 2,5-Piperazinediones with an Anneled Azetidine Ring; Synthesis and Infrared Spectra. *Collect. Czechoslov. Chem. Commun.* **1973**, *38* (7), 1957–1970. https://doi.org/10.1135/cccc19731957.
